# Diverging Food Web Functioning Around Southampton Island, Nunavut: The Influence of Primary Production Supply and Bathymetry

**DOI:** 10.1002/ece3.73448

**Published:** 2026-04-15

**Authors:** Rémi Amiraux, C. J. Mundy, Marie Pierrejean, Philippe Massicotte, Steven H. Ferguson, Aaron T. Fisk, Les N. Harris, Kevin J. Hedges, Kelsey F. Johnson, Andrea Niemi, Bruno Rosenberg, Wesley R. Ogloff, Cortney A. Watt, David J. Yurkowski

**Affiliations:** ^1^ Laboratoire de Recherche International Takuvik CNRS, Université Laval, Sorbonne Université Québec Québec Canada; ^2^ Centre for Earth Observation Science University of Manitoba Winnipeg Manitoba Canada; ^3^ Freshwater Institute, Arctic Region, Fisheries and Oceans Canada Winnipeg Manitoba Canada; ^4^ Department of Biological Sciences University of Manitoba Winnipeg Manitoba Canada; ^5^ Great Lakes Institute for Environmental Research University of Windsor Windsor Ontario Canada

**Keywords:** benthic, carbon flux, diversity, functional traits, highly branched isoprenoids δ^13^C, highly branched isoprenoids δ^15^N, Hudson Bay, pelagic, Southampton Island, stable isotopes

## Abstract

The structure and functioning of Arctic marine food webs are shaped by the origin (ice‐ or pelagic‐derived), quantity, and quality of primary production, which are influenced by environmental factors. While pan‐Arctic variability has been widely studied, spatial differences at smaller scales (~10–500 km) remain poorly understood. In this study, we examined north–south spatial variability around Southampton Island in the Hudson Bay (Nunavut) focusing on (i) vertical trophic structure, (ii) the relative contributions of ice algae and phytoplankton‐derived carbon to the food web and (iii) benthic functional diversity. We measured bulk stable carbon and nitrogen isotopes as well as highly branched isoprenoids in samples belonging to 149 species, including invertebrates, fishes, and marine mammals. We found marked contrasts between the northern and southern food webs, reflecting different environmental conditions. The north was characterized by deeper depths (mean of 132.0 m), with relatively lower ice algae production relative to phytoplankton, a narrow δ^13^C range, low sympagic carbon (mean of 13.5% ± 10.7%), and dominance of filter feeders. As a result, the northern benthic sub‐web relied mainly on phytoplankton and occupied four trophic levels, including top predator sea stars. In contrast, the south was shallower (mean 60.7 m), with higher ice algae input, a broader δ^13^C range, higher sympagic carbon (35.1% ± 19.7%), a benthic sub‐web occupying only three trophic levels, and a dominance of deposit feeders. Here, trophic truncation among benthic species likely represents relatively stronger top‐down control by walruses, a benthivorous predator that specializes on large bivalves, facilitated by a shallower bathymetry and is considered prime walrus habitat. However, this selective pressure fosters benthic functional trait richness by releasing non‐target species, in conjunction with a diversified food supply from both ice algae and phytoplankton. These results demonstrate how small‐scale spatial variation in depth and primary production sources can restructure Arctic food webs, with implications for ecosystem functioning under changing ice conditions.

## Graphical abstract

Southampton Island hosts two distinct benthic sub‐webs, north and south. North is deeper, phytoplankton‐driven; South is shallower, more ice‐algae driven. North benthos: bottom‐up control, four trophic levels including apex predators. South benthos: walrus top‐down control, trophic truncation to three levels. Joint top‐down control and mixed food supply boost benthic functional diversity.

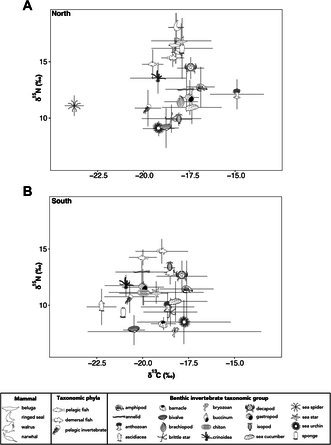

## 1 Introduction

Climate warming is forcing rapid changes to Canada's Arctic marine icescape and its associated ecosystems, with Hudson Bay being a region of particular concern (Amiraux et al. 2022; Gupta et al. 2022; Lukovich et al. 2021; Regehr et al. 2007). Sea ice plays a central role in polar marine ecosystems as it drives the phenology of primary producers that constitute the base of marine food webs. The energy from primary producers then transfers up through successive trophic levels in the pelagic and benthic environments from zooplankton or zoobenthos to fish and to top predators including seabirds and marine mammals, with each level dependent on preceding levels for energy (Amiraux, Mundy, et al. 2023; Amiraux, Yurkowski, et al. 2023; Koch et al. 2023; Lindemann 1942). Food chain length, the number of trophic levels within a food web or sub‐web, is mediated by several environmental factors including ecosystem size, productivity, disturbance, and omnivory, which is widespread in marine systems and occurs when a predator consumes species from multiple trophic levels (Post 2002a; Takimoto and Post 2013). Both primary productivity and level of omnivory can act synergistically together, where higher primary productivity levels can increase the level of omnivory, which then shortens the food chain (Lerner et al. 2022).

In surface marine environments of the Arctic, microalgal production includes two consecutive pulses of marine autotrophs: sea‐ice algae and phytoplankton (Loose et al. 2011; Wassmann and Reigstad 2011). An additional autotrophic source is benthic micro‐ and macroalgal production, although it is restricted to shallow (< 50 m) zones where enough light reaches the ocean floor (Castro de la Guardia et al. 2023; Filbee‐Dexter et al. 2019; Glud et al. 2009). In early spring, increasing irradiance enables ice algae to grow (Mundy and Meiners 2021). Later in the season, when snow and sea ice melt, a phytoplankton bloom develops and follows the ice retreat (Barbedo et al. 2020; Matthes et al. 2021). Measurements of phytoplankton productivity far outnumber those for ice algae; however, it has been estimated that ice algae contribute 1%–26% of the total primary production across Arctic shelves (Legendre et al. 1992; Payne et al. 2021), while in the central Arctic Ocean, this contribution can reach 57%–83% (Boetius et al. 2013; Gosselin et al. 1997). Because of their relatively high sinking rates and nutritional quality, ice algae play an important role in sympagic‐pelagic‐benthic coupling (Amiraux, Archambault, et al. 2021; Amiraux, Mundy, et al. 2023; Niemi et al. 2024). This, in turn, supports both the benthic and pelagic sub‐webs, which together form the marine food web (Amiraux, Yurkowski, et al. 2023). These spatial asynchronies between benthic and pelagic energy pathways are coupled by mobile, higher trophic level consumers that can alter their feeding behaviors relative to resource density leading to increased food web connectance and structure (Rooney et al. 2006; Rooney and McCann 2012). These habitat coupling species are generally near‐apex and apex predators who also play an important role in structuring Arctic food webs through strong top‐down trophic control, which is a key determinant of polar ecosystem structure and function (Boyce et al. 2015).

However, with a decrease in sea ice extent and duration, anthropogenic climate change threatens ice algal productivity while promoting increased net phytoplankton and macroalgal productivity, which can impact the level of omnivory and trophic control of the Arctic food web (Goldsmit et al. 2021; Kortsch et al. 2012; Lewis et al. 2020; Weslawski et al. 2010). To understand the impacts of predicted productivity shifts, it is critical to assess the dependency of Arctic food webs on sea ice and its resources, and how their responses vary spatially.

Analyses of stable carbon (δ^13^C) and nitrogen (δ^15^N) isotopes, together with highly branched isoprenoid (HBI) applied to biological samples, provide inferences about the dependency of Arctic consumers on ice‐associated and pelagic resources throughout the food chain. δ^15^N reflects the relative trophic level of consumers (i.e., diet) while δ^13^C traces the primary energy source (i.e., sympagic, pelagic or benthic‐derived energy; France 1995; Minagawa and Wada 1984). However, the close δ^13^C values between macroalgae and ice algae, as well as between phytoplankton and terrestrial organic matter may, in some cases, lead to ambiguous and limited quantitative estimates (McMeans et al. 2013). To provide an unequivocal and quantitative relative contribution of ice algae and phytoplankton that feed consumers and the entire food web, HBI‐based methods have recently been widely employed (Brown et al. 2014; Brown and Belt 2017; Brown 2018; Brown et al. 2018). Among HBIs, the monoene IP_25_ (Ice Proxy with 25 carbon atoms) is a biomarker of ice algae, while the triene HBI reflects phytoplankton production (Belt 2018). Consequently, numerous HBI‐based indices have been developed to estimate the relative contributions of ice algae and phytoplankton to consumers and throughout the food web (Brown et al. 2018; Cusset et al. 2019; Koch et al. 2023; Yunda‐Guarin et al. 2020), without interference from other organic matter such as terrestrial inputs or macroalgae. For complex coastal habitats, particularly in the Arctic, which include many species with different life histories and varied diets that may also shift seasonally, biomarker approaches provide novel insights into food web structures that would otherwise be impossible to obtain (e.g., McGovern et al. 2018; Mohan et al. 2016).

**FIGURE 1:**
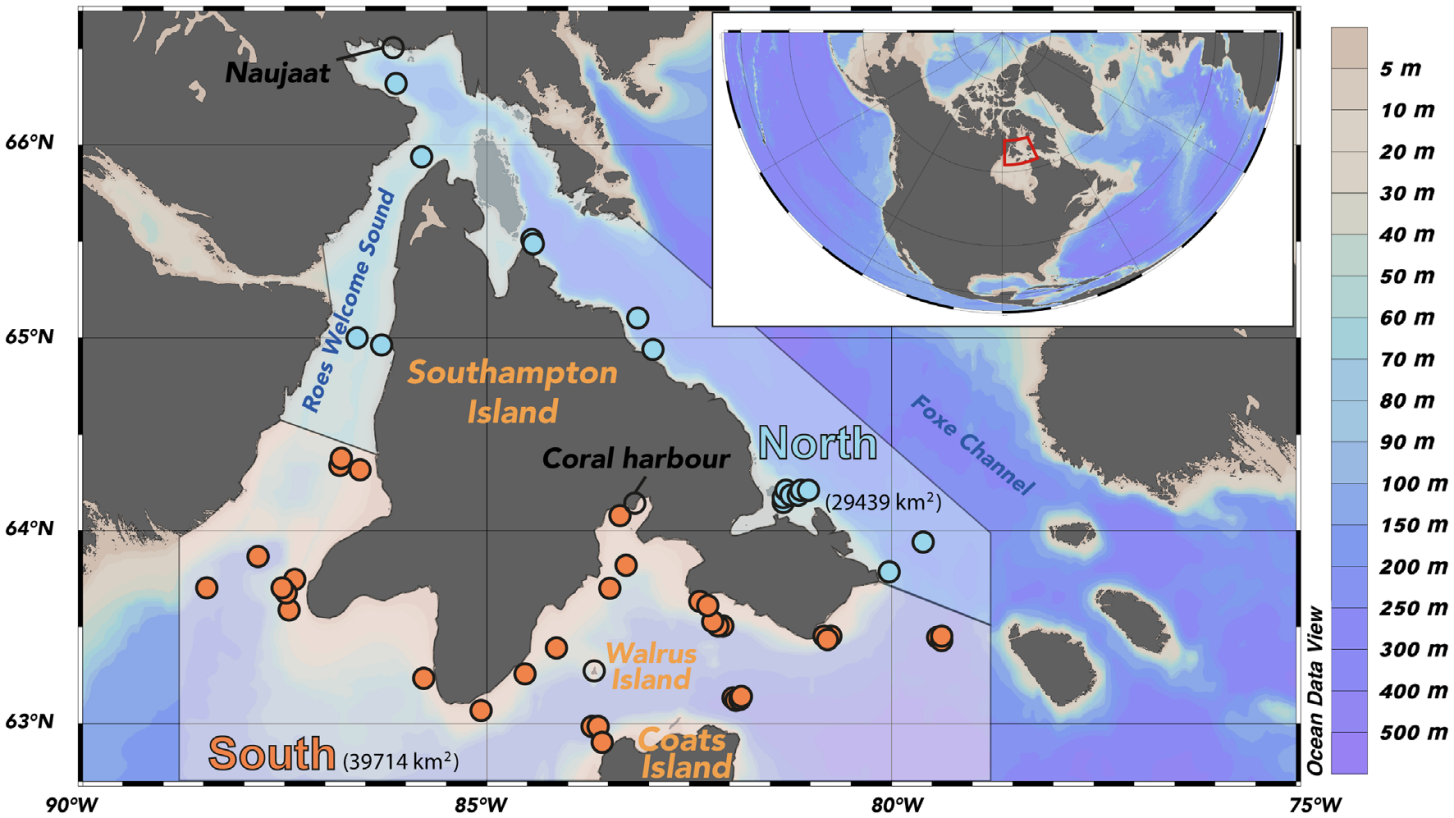
Map of the study area around Southampton Island in Hudson Bay, with locations of the northern and southern areas and the stations investigated.

The marine region around Southampton Island, northwest Hudson Bay (Nunavut, Figure 1), is an interesting marine environment where nutrient‐replete deep water from Foxe Basin is mixed along constricted waterways to the northwest, supporting high phytoplankton production. In the southern zone, however, water masses originating in the northern region (Ridenour et al. 2019) are not as nutrient‐rich, and the water‐column tends to be dominated by strong surface stratification, particularly during summer, which can limit primary production (Kitching et al. 2026). Although Hudson Bay is generally viewed as oligotrophic, multiple lines of evidence support the notion of high biological productivity, specifically benthic productivity, in the Southampton Island region (Castro de la Guardia et al. 2023; Filbee‐Dexter et al. 2022). This region encompasses one of Canada's largest summer and winter aggregations of numerous Arctic marine mammal species (Loewen et al. 2020). Because of the multiple ecosystem services provided by this biological hotspot (Loewen et al. 2020), this region has supported local human habitation for millennia, with confirmed Dorset, Thule, and Sadlermiut occupation sites (Clark 1980; Collins 1956; McGhee 1970). Its marine resources remain critical to the subsistence economy of local communities today. For these reasons, this region has been identified as an Area of Interest for Marine Protected Area (MPA) designation by Fisheries and Oceans Canada (DFO 2020; Loewen et al. 2020).

In a previous study, we examined the entire marine food web and discovered that sympagic carbon, defined as carbon produced in the sympagic (sea‐ice) compartment, is crucial for sustaining this ecosystem at every taxonomic level, including marine mammals, which contain 53.3% ± 22.2% of ice algae‐derived carbon (Amiraux, Mundy, et al. 2023). However, there are significant environmental differences—that is, water masses, depth, sea ice—between the northern and southern marine areas of this island that could lead to divergence in food web functioning, that is, the combination of functional diversity, carbon source use, and consumer trophic positions, between geographic areas. The northern region features a polynya and hosts summering narwhals ( *Monodon monoceros* ) from the Hudson Bay population (approximately 19,200 individuals; Watt and Hornby 2020), along with several other marine mammal species such as beluga whales, ringed seals ( *Pusa hispida* ) and bearded seals ( *Erignathus barbatus* ). In contrast, marine waters at the southern end of Southampton Island support substantially lower densities of belugas, narwhals, and ringed seals than in the north, while large aggregations of Atlantic walrus ( *Odobenus rosmarus rosmarus* ) are present on several known haul outs in the area, including the aptly named Walrus Island in Fisher Strait between Southampton Island and Coats Island (Hammill et al. 2016).

Spatial heterogeneity in water masses, bathymetry and marine mammal densities and diversity over relatively small scales (< 200 km) between northern and southern Southampton Island likely indicates that the food resources available to these species, and consequently the structure and functioning of these systems, may vary between the northern and southern regions of the island. Therefore, our goal is to demonstrate how local‐ and regional‐scale environmental heterogeneity can shape trophic position and carbon source use of consumers inhabiting a coastal Arctic food web. Given the environmental and ecological contrasts around Southampton Island, we hypothesize there will be a significant difference in benthic versus pelagic sub‐webs and their carbon source between the north and south of Southampton Island. To provide important insights into Arctic coastal dynamics and inform conservation and management initiatives for these systems, it is thus essential to better characterize how environmental factors, such as primary productivity supply and taxonomic source (indicated by stable isotopes and HBIs) as well as bathymetry, shape several food web properties in coastal ecosystems.

## 2 Materials and Methods

### 2.1 Sample Collection

Benthic and pelagic invertebrates, fishes, and marine mammals were sampled in summer 2016, 2018, and 2019 in the marine waters around Southampton Island, Nunavut, Canada (Figure 1). This study uses the same dataset as Amiraux, Mundy, et al. (2023), where sampling and analytical methods are detailed. Here, we group species into two compartments where the benthic compartment is composed of benthic invertebrates and the pelagic compartment is composed of pelagic invertebrates, demersal and pelagic fishes, and marine mammals. We also subdivided the study area into northern and southern zones by adapting the water mass classification proposed by Kitching et al. (2026) to better reflect both water mass properties and observed patterns in marine mammal diversity. Among them, the higher density of walrus in the south and, conversely, a greater presence of narwhals and belugas ( *Delphinapterus leucas* ) in the north, particularly in Roes Welcome Sound, Naujaat (Repulse Bay), and Foxe channel (Loewen et al. 2020; Watt and Hornby 2020). Although Southampton Island spans ~42,000 km^2^, with a maximum north–south distance of ~335 km and an east–west width of ~350 km, its triangular shape means that the northern and southern marine areas are often separated by less than 200 km (Figure 1). Southampton Island lies at the junction of Hudson Bay and Foxe Basin, and the northern and southern marine sectors are connected through a narrow system of straits. The north of the island exchanges water with Foxe Basin via Repulse Bay and Frozen Strait, while the south opens into Hudson Bay through Roes Welcome Sound. These passages experience strong tidal and wind‐driven mixing, which promotes the exchange of water masses, nutrients, and plankton communities between the two regions (DFO 2020). Of the sampled benthic invertebrates reported by Amiraux, Mundy, et al. (2023)—97 species across 19 taxonomic groups—339 were from the north and 541 from the south. Functional traits of benthic organisms were retrieved from the World Register of Marine Species (WoRMS) database. Pelagic invertebrates—20 species in eight taxonomic groups—totaled 122 in the north and 181 in the south. Among demersal fishes (20 species), 61 muscle and eight liver samples were processed from the north and 244 muscle and 18 liver samples from the south. Among pelagic fishes (five species), we processed 77 muscle and 72 liver samples from the north and 49 muscle and six liver samples from the south. Of the marine mammal species collected, 68 muscle and 64 liver samples belonging to ringed seal, narwhal, and beluga were from the north, while four muscle and four liver samples from Atlantic walrus were from the south.

### 2.2 Bathymetry and Sea Ice Concentration

To delineate the spatial extent of the study area for sea ice concentration, polygons were defined based on the sampling locations (see Figure 1). Within these polygons, sea ice concentration data were extracted from AMSR2 observations provided by the University of Bremen, covering June to August from 2016 to 2019. A threshold of 15% sea ice concentration was applied to classify each 3 km pixel as either ice‐covered or open water. Bathymetry data (in meters) for the sampling area and sites were extracted from the General Bathymetric Chart of the Oceans 2023 grid (doi: 10.5285/f98b053b‐0cbc‐6c23‐e053‐6c86abc0af7b). All bathymetry and sea ice data were processed in R version 4.4.2 (R Core Team 2024) using the ‘terra’ package (Hijmans et al. 2026).

### 2.3 Biomarker Analysis

The analysis of bulk stable isotope and HBI, as well as the calculation of trophic position (TP) and sympagic carbon percent (SC%), are described in Amiraux, Mundy, et al. (2023). Briefly, lyophilized and homogenized tissues underwent lipid extraction using a chloroform: methanol (2:1) solvent following a modified Bligh and Dyer (1959) protocol. Subsamples of the defatted material were used for δ^13^C, δ^15^N analysis using a Delta V Advantage Mass spectrometer (Thermo Finnigan, San Jose, CA, USA) coupled to a Costech 4010 Elemental Combustion system (Costech, Valencia, CA, USA) and a ConFlo IV gas interface. TP was calculated using a one‐source model (Post 2002b) with *Calanus hyperboreus* (TP = 2) as the baseline. Although benthic taxa would typically require a benthic primary consumer such as a sea urchin or bivalve as the reference, the δ^15^N values of these benthic grazers were comparable to that of the pelagic copepod, justifying its use as a common baseline across taxa (mean ± SD of 9.8‰ ± 0.7‰, 8.5‰ ± 1.7‰, 8.7‰ ± 1.6‰ for copepod, bivalve, and sea urchin respectively). A trophic enrichment factor (Δ^15^N) of 3.4‰ was applied (Post 2002b) to the measured δ^15^N value of each consumer, except for piscivores, for which 2.4‰ was used (Hobson et al. 1996). Lipid extracts from the isotope preparation were repurposed for HBI analysis: following saponification, HBIs were extracted with hexane and analyzed by GC–MS in SIM mode (Belt et al. 2012). The relative abundances of IP25, HBI II, and HBI III were used to compute the H‐Print index, from which SC% was derived using a validated calibration equation (Brown et al. 2014; Brown and Belt 2017).

### 2.4 Statistical Analysis

Because both stable isotopes were normally distributed, one‐way ANOVAs were performed to determine if δ^13^C and δ^15^N were different between the two regions. Given the non‐normal distribution of TP and SC% among consumers, as well as comparisons of the environmental variables, we employed non‐parametric Wilcoxon rank‐sum tests to determine significant differences between the northern and southern study regions. Linear regressions were conducted between log₁₀‐transformed sympagic carbon percentages and the calculated trophic positions. All statistical analyses were performed using R software version 4.4.2 (R Core Team 2024).

## 3 Results

The northern region is deeper (mean ± SD of 132.0 ± 98.1 m vs. 60.7 ± 50.3 m, respectively) and has a shorter ice‐free season (mean ± SD of 111.3 ± 10.6 days vs. 145.3 ± 11.7 days, respectively; Figure S1). In the north, benthic invertebrates were sampled at 19 stations, where 16 taxonomic groups spanning 55 species were identified. In the South, benthic sampling at 25 stations yielded 18 taxonomic groups spanning 62 species (Tables 1 and 2, Tables S1 and S2). In the north, pelagic invertebrates consisted of eight taxonomic groups spanning 14 species, while in the south it consisted of seven taxonomic groups spanning 17 species. Demersal fishes included eight species in the north and 18 in the south. Pelagic fishes included the same three species in both regions. Marine mammal sampling in the northern region included belugas, narwhals, and ringed seals, whereas only walrus were sampled in the southern region (Tables 1 and 2, Tables S1 and S2).

**TABLE 1:** Carbon and nitrogen stable isotope ratio (δ^13^C and δ^15^N), trophic position (TP), and sympagic carbon (%) of marine food web organisms at the phylum level in north Southampton Island.

Taxonomic phyla	Taxonomic group	Tissue	*n* sampled	*n*	δ13C (‰)	δ15N (‰)	TP	*n*	Sympagic C (%)
Benthic invertebrate		Whole organism, soft part, piece of muscle	617	339	−17.1 ± 1.9	12.8 ± 2.8	3.0 ± 0.8	205	13.5 ± 10.7
	Amphipod	Whole	87	31	−16.9 ± 0.8	12.8 ± 1.8	3.0 ± 0.5	14	14.7 ± 9.3
	Anthozoan	Whole	26	22	−14.9 ± 1.5	12.1 ± 1.3	2.8 ± 0.4	14	10.9 ± 7.8
	Bivalve	Soft part	46	26	−18.8 ± 0.7	9.1 ± 1.8	1.9 ± 0.5	4	13.6 ± 3.3
	Brachiopod	Soft part	7	6	−17.9 ± 0.9	11.5 ± 0.5	2.6 ± 0.1	‐^a^	—
	Brittle star	Piece	17	8	−18.5 ± 1.6	10.0 ± 1.3	2.1 ± 0.4	6	14.0 ± 6.3
	Crinoid	Piece	14	11	−19.3 ± 1.5	13.6 ± 1.4	3.2 ± 0.4	12	10.1 ± 3.1
	Decapod	Muscle	168	64	−17.4 ± 0.7	14.4 ± 1	3.4 ± 0.3	70	6.0 ± 3.3
	Gastropod	Soft part	29	28	−17.5 ± 0.5	11.8 ± 1.5	2.7 ± 0.5	3	9.9 ± 0.4
	Isopod	Whole	33	14	−18.1 ± 0.7	9.9 ± 0.6	2.1 ± 0.2	9	23.9 ± 3.1
	Sea cucumber	Whole	22	17	−17.4 ± 1.6	11.0 ± 1.5	2.4 ± 0.4	6	6.6 ± 4.2
	Sea spider	Piece	32	11	−23.8 ± 0.5	11.1 ± 0.9	2.5 ± 0.3	10	22.4 ± 11.8
	Sea star	Piece	27	21	−17.9 ± 1.6	16.9 ± 1.6	4.2 ± 0.5	15	16.4 ± 9.8
	Sea urchin	Piece	24	12	−19.2 ± 0.9	9.1 ± 0.7	1.9 ± 0.2	12	8.0 ± 4
	Sponge	Whole	37	35	−17.9 ± 1.7	16.2 ± 2.7	4.0 ± 0.8	19	29.1 ± 10.4
	Worms	Whole	46	33	−17.6 ± 1.1	12.6 ± 2	2.9 ± 0.6	11	30.1 ± 9.8
Pelagic invertebrate		Whole	256	122	−19.8 ± 0.7	10.9 ± 1.6	2.4 ± 0.5	29	12.3 ± 17.8
Demersal fish		Muscle	61	61	−18.5 ± 0.9	14.4 ± 1.5	3.4 ± 0.4	8	21.5 ± 9.3
Pelagic fish		Muscle	114	77	−19.9 ± 0.7	14.1 ± 1.5	3.3 ± 0.4	72	7.4 ± 7.7
Marine mammal		Muscle		68	−18.2 ± 0.3	16.8 ± 1.5	4.5 ± 0.6	64	53.5 ± 24.6

^a^ Not analyzed.

**TABLE 2:** Carbon and nitrogen stable isotope ratio (δ^13^C and δ^15^N), trophic position (TP), and sympagic carbon (%) of marine food web organisms at the phylum level in south Southampton Island.

Taxonomic phyla	Taxonomic group/species	Tissue	*n* sampled	*n*	δ13C (‰)	δ15N (‰)	TP	*n*	Sympagic C (%)
Benthic invertebrate		Whole organism, soft part, piece of muscle	1253	541	−18.4 ± 2.3	11.3 ± 2.2	2.5 ± 0.6	260	35.1 ± 19.7
	Amphipod	Whole	92	40	−17.6 ± 1.5	11.4 ± 2.5	2.6 ± 0.7	16	26.1 ± 17.9
	Anthozoan	Whole	11	9	−18.6 ± 1.5	9.8 ± 1.3	2.1 ± 0.4	2	10.1 ± 9.7
	Ascidiacea	Whole	49	41	−22.2 ± 0.8	9.8 ± 1.6	2.1 ± 0.5	8	26.7 ± 3.2
	Barnacle	Soft part	36	25	−18.9 ± 0.7	8.3 ± 0.6	1.7 ± 0.2	11	58.1 ± 3.5
	Bivalve	Soft part	27	22	−20.5 ± 0.6	7.8 ± 1.3	1.5 ± 0.4	‐^a^	—
	Brittle star	Piece	205	30	−18.5 ± 1.5	9.4 ± 1.3	2.0 ± 0.4	20	40.2 ± 18.3
	Bryozoan	Piece	9	2	−18.3 ± 4.7	7.6 ± 0.2	1.4 ± 0.1	9	44.9 ± 14.7
	Chiton	Soft part	19	10	−19.9 ± 1.3	11.0 ± 1.1	2.4 ± 0.3	—	—
	Crinoid	Piece	14	10	−20.9 ± 1.2	11.7 ± 1.0	2.6 ± 0.3	13	29.2 ± 26.2
	Decapod	Muscle	579	216	−17.9 ± 1.4	12.6 ± 1.4	2.9 ± 0.4	124	31.4 ± 19.1
	Gastropod	Soft part	87	59	−20.0 ± 1.1	11.6 ± 1.6	2.6 ± 0.5	20	39.0 ± 19.6
	Isopod	Whole	13	10	−18.5 ± 0.3	13.3 ± 0.5	3.1 ± 0.1	—	—
	Sea cucumber	Whole	9	7	−18.2 ± 1.7	10.3 ± 1.8	2.2 ± 0.5	—	—
	Sea star	Piece	24	12	−19.3 ± 2.6	11.1 ± 1.0	2.5 ± 0.3	15	34.7 ± 11
	Sea urchin	Piece	34	19	−17.8 ± 2.6	8.5 ± 2	1.7 ± 0.6	12	52.6 ± 22
	Sponge	Whole	2	1	−21.0	9.3	1.9	2	39.3 ± 18.5
	Worms	Whole	39	28	−20.0 ± 1.4	12.9 ± 1	3.0 ± 0.3	8	44.8 ± 19
Pelagic invertebrate		Whole	181	181	−20.7 ± 1.4	10.7 ± 1.4	2.4 ± 0.4	99	26.0 ± 22.0
Demersal fish		Muscle	244	244	−18.9 ± 1.5	14.3 ± 1.2	3.4 ± 0.4	18	11.9 ± 11.3
Pelagic fish		Muscle	49	44	−20.0 ± 0.8	14.0 ± 0.9	3.3 ± 0.3	6	10.1 ± 2.9
Marine mammal		Muscle	4	4	−18.5 ± 0.3	12.9 ± 1.0	3.0 ± 0.3	4	44.5 ± 7.8

^a^ Not analyzed.

On average, both δ^13^C and δ^15^N values were higher in the northern Southampton Island marine food web compared to the southern region, with δ^13^C values of −18.5‰ ± 1.6‰ in the north compared to −19.2‰ ± 1.8‰ in the south, and δ^15^N values of 13.6‰ ± 2.9‰ in the north compared to 12.2‰ ± 2.3‰ in the south. As these standard deviations suggest, the northern food web exhibits greater variability in δ^15^N values among different phyla and taxonomic groups, while the southern food web shows greater variability in δ^13^C values. One‐way ANOVA revealed a small but significant effect size of region on δ^13^C values (*F*(1, 1791) = 81.24, *p* < 0.001, *η* ^2^ = 0.04, 95% CI [0.03, 1.00]) and a medium‐size effect of δ^15^N values (*F*(1, 1791) = 130.24, *p* < 0.001, *η* ^2^ = 0.07, 95% CI [0.05, 1.00]). This regional effect was also significant within all taxonomic phyla except pelagic fish. These results indicate meaningful spatial differences in isotopic composition.

**FIGURE 2:**
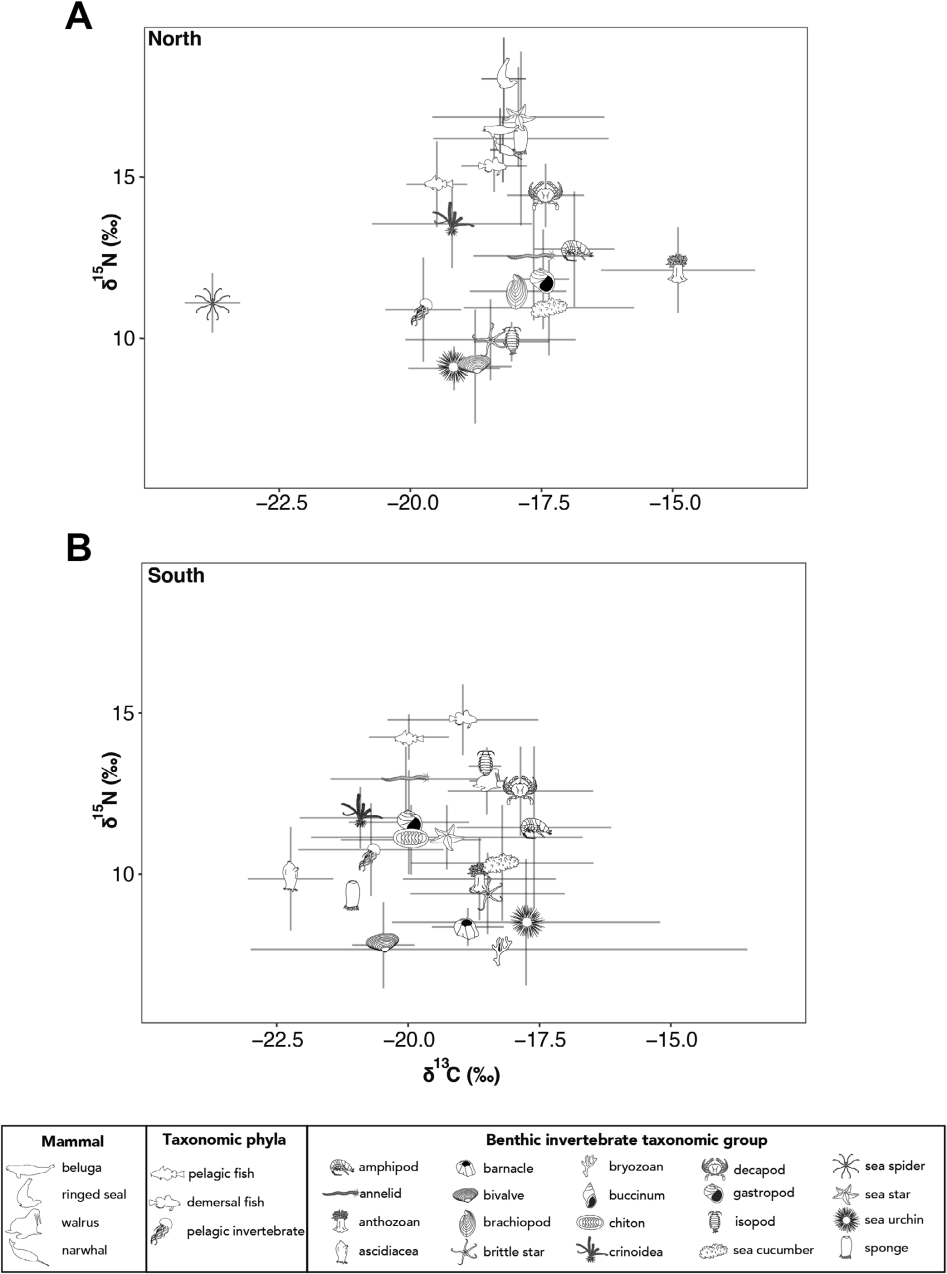
Relationship between carbon and nitrogen stable isotope ratios (δ^13^C and δ^15^N; ‰) for organisms aggregated as benthic invertebrate groups and pelagic invertebrate, fish, and marine mammal phyla in the (A) northern and (B) southern Southampton Island marine food webs.

Across the northern food web, δ^13^C ranged from −23.9‰ ± 0.2‰ in the sea spider *Boreonymphon abyssorum* to −14.9‰ in the Anthozoan *Gersemia* spp. The δ^15^N, values in the north region ranged from 7.5‰ in the benthic bivalve *Ciliatocardium ciliatum* to 19.2‰ in an asteroidea (seastar) (Table S1). Across the southern Southampton Island marine food web, δ^13^C ranged from −23.6‰ ± 0.5‰ in the pelagic invertebrate *Pteropoda* to −14.9‰ ± 1.1‰ in the benthic Decapod *Argis dentata* . The δ^15^N values in the south region ranged from 6.5‰ ± 0.2‰ in the benthic bivalve *Crenella* sp. to 16.0‰ ± 0.5‰ in the demersal fish *Eumesogrammus praecisus* (Table S2). Benthic and pelagic invertebrates showed similar δ^15^N values between the north and south (benthic: 12.8‰ ± 2.8‰ vs. 11.3‰ ± 2.2‰; pelagic: 10.9‰ ± 1.6‰ vs. 10.7‰ ± 1.4‰; Tables 1 and 2) but differed in δ^13^C values (benthic: −17.1‰ ± 1.9‰ vs. −18.4‰ ± 2.3‰; pelagic: −19.8‰ ± 0.7‰ vs. −20.7‰ ± 1.4‰; Tables 1 and 2). Like benthic invertebrates, pelagic invertebrates exhibited greater variability in δ^15^N and lower variability in δ^13^C in the north compared to the south. On average, pelagic and benthic invertebrates were lower in δ^15^N compared to fishes and marine mammals (Tables 1 and 2; Figure 2). However, likely due to the wide variety of taxonomic groups and species within the invertebrates, it is not surprising that some benthic invertebrate species have higher δ^15^N values than some fish or even marine mammals (Figure 2). Indeed, in the northern area, the highest average δ^15^N value was observed in a sea star as well as in marine mammals (16.8‰ ± 1.5‰), while in the southern food web, the highest average δ^15^N value was observed in demersal and pelagic fishes (14.3‰ ± 1.2‰ and 14.0‰ ± 0.9‰, respectively) as well as in a parasitic benthic isopod, *Bopyroides hippolytes* (13.3‰ ± 0.5‰; Figure 2). On average, pelagic and demersal fishes had similar isotopic values regardless of whether they were collected in the northern or southern region. For demersal fishes, δ^13^C was −18.5‰ ± 0.9‰ in the north and −18.9‰ ± 1.5‰ in the south, while δ^15^N was 14.4‰ ± 1.5‰ in the north and 14.3‰ ± 1.2‰ in the south. For pelagic fishes, δ^13^C was −19.9‰ ± 0.7‰ in the north and −20.0‰ ± 0.8‰ in the south, with δ^15^N values of 14.1‰ ± 1.5‰ in the north and 14.0‰ ± 0.9‰ in the south.

**FIGURE 3:**
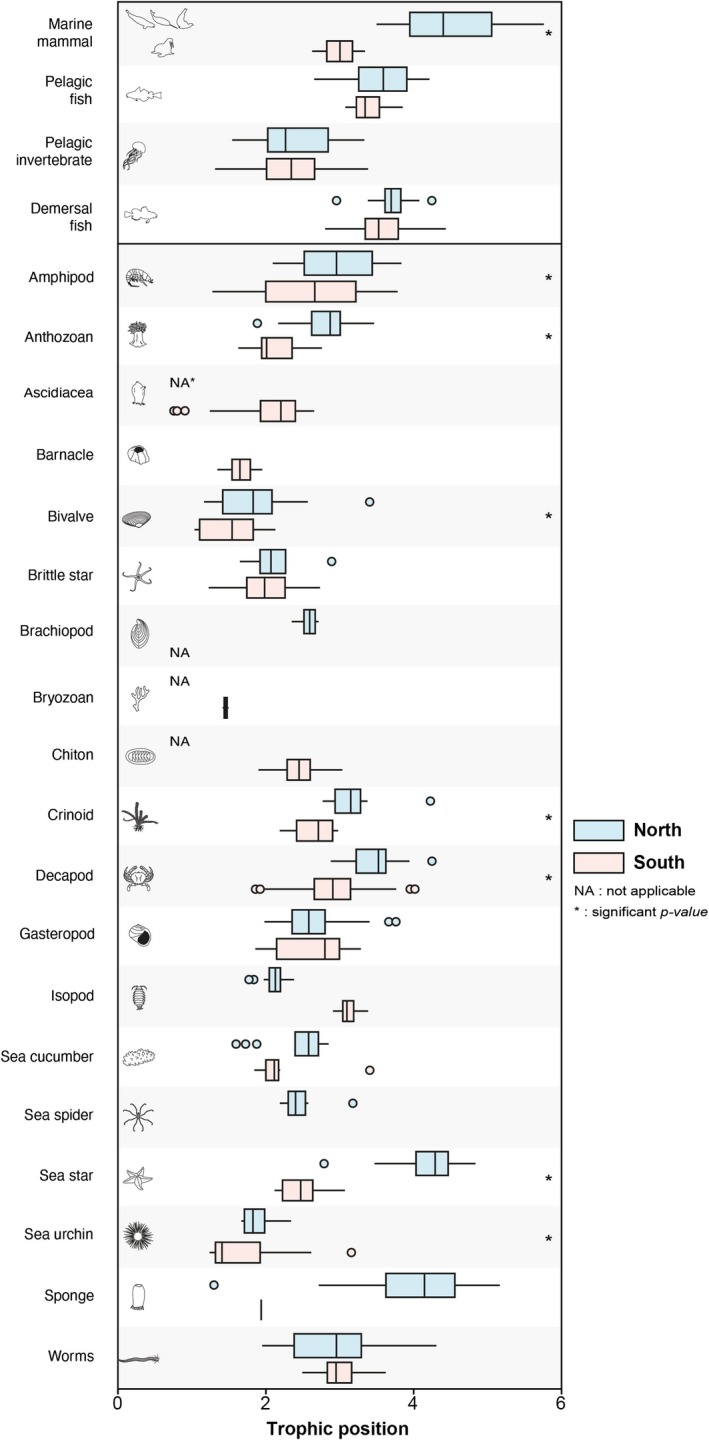
Comparison of trophic positions (TP) between northern and southern Southampton Island marine food webs. Data shown for benthic invertebrate taxonomic groups and pelagic phyla. Asterisks (*) indicate statistically significant differences (*p* ≤ 0.05) based on Wilcoxon tests (see Table S3 for detailed results).

In the northern region, average TP ranged from 1.4 in the bivalve *Ciliatocardium ciliatum* to 4.8 in an asteroidea (sea star) and in ringed seals (4.8 ± 0.5) (Table S1). In the south, average TP ranged from 1.1 ± 0.1 in the bivalve *Crenella* sp. to 3.7 ± 0.1 in demersal fish *Myoxocephalus scorpius* (Table S2). In ascending order in the northern region, pelagic invertebrates occupied a TP of 2.4 ± 0.5, benthic invertebrates of 3.0 ± 0.8 [1.4–4.8], pelagic fishes of 3.3 ± 0.4, demersal fishes of 3.4 ± 0.4 and marine mammals (beluga, narwhal, ringed seal) of 4.5 ± 0.6 (Table 1). In the southern region, except for marine mammals, the different phyla investigated occupied the same trophic positions as in the northern region, with pelagic invertebrates at a TP of 2.4 ± 0.4, benthic invertebrates at 2.5 ± 0.6 [1.1–3.9], pelagic fishes at 3.3 ± 0.3, demersal fishes at 3.4 ± 0.4, and marine mammals (walrus) at 3.0 ± 0.3 (Table 2). More than 50% of the benthic taxonomic groups had a significantly higher trophic position in the north compared to the south (Figure 3; Table S3A).

**FIGURE 4:**
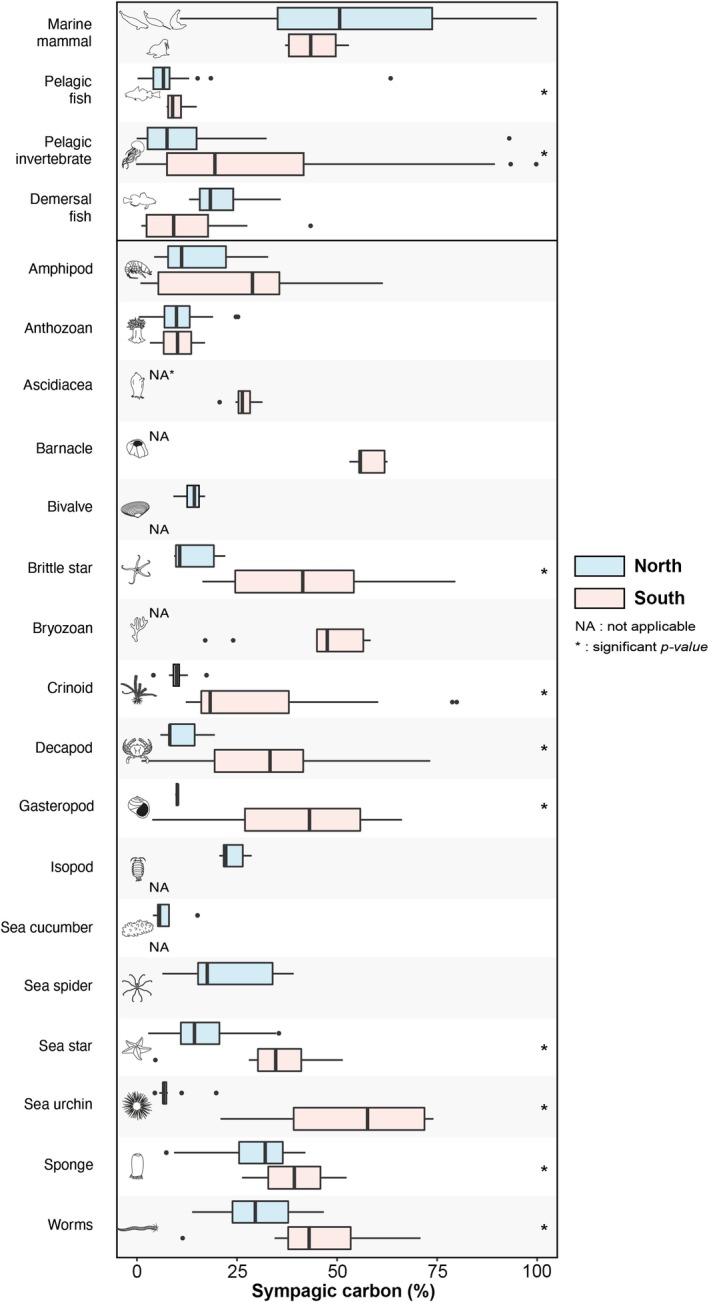
Sympagic carbon (%), calculated from HBI analysis, of organisms in the northern and southern Southampton Island marine food webs at the benthic invertebrate taxonomic group and pelagic phylum level. Asterisks (*) denote statistically significant differences (Wilcoxon test, *p* ≤ 0.05; see Table S3 for detailed results).

In all species investigated in the northern region, SC% ranged from 1.4% in the benthic anthozoan *Actiniaria* sp. to 60.7% ± 22.8% in ringed seals (Table S1). In the southern region, SC% ranged from 3.2% in the benthic anthozoan *Nephtheidae* to 94.8% ± 7.3% in the pelagic copepod *Calanus hyperboreus* (Table S2). In the northern region, the average SC% was 13.5% ± 10.7% for benthic invertebrates, 12.3% ± 17.8% for pelagic invertebrates, 21.5% ± 9.3% for demersal fishes, 7.4% ± 7.7% for pelagic fishes, and 53.5% ± 24.6% for marine mammals (Table 1). In the southern region, the average SC% was 35.1% ± 19.7% for benthic invertebrates, 26.0% ± 22.0% for pelagic invertebrates, 11.9% ± 11.3% for demersal fishes, 10.1% ± 2.9% for pelagic fishes, and 44.5% ± 7.8% for marine mammals (Table 2). With the exception of demersal fishes and marine mammals, most of the benthic and pelagic taxonomic groups have a significantly lower SC% in the north compared to the south (Figure 4; Table S3B). The relationship between sympagic carbon percentage and trophic position in benthic invertebrates showed no significant trend across regions (Figure S2).

**FIGURE 5:**
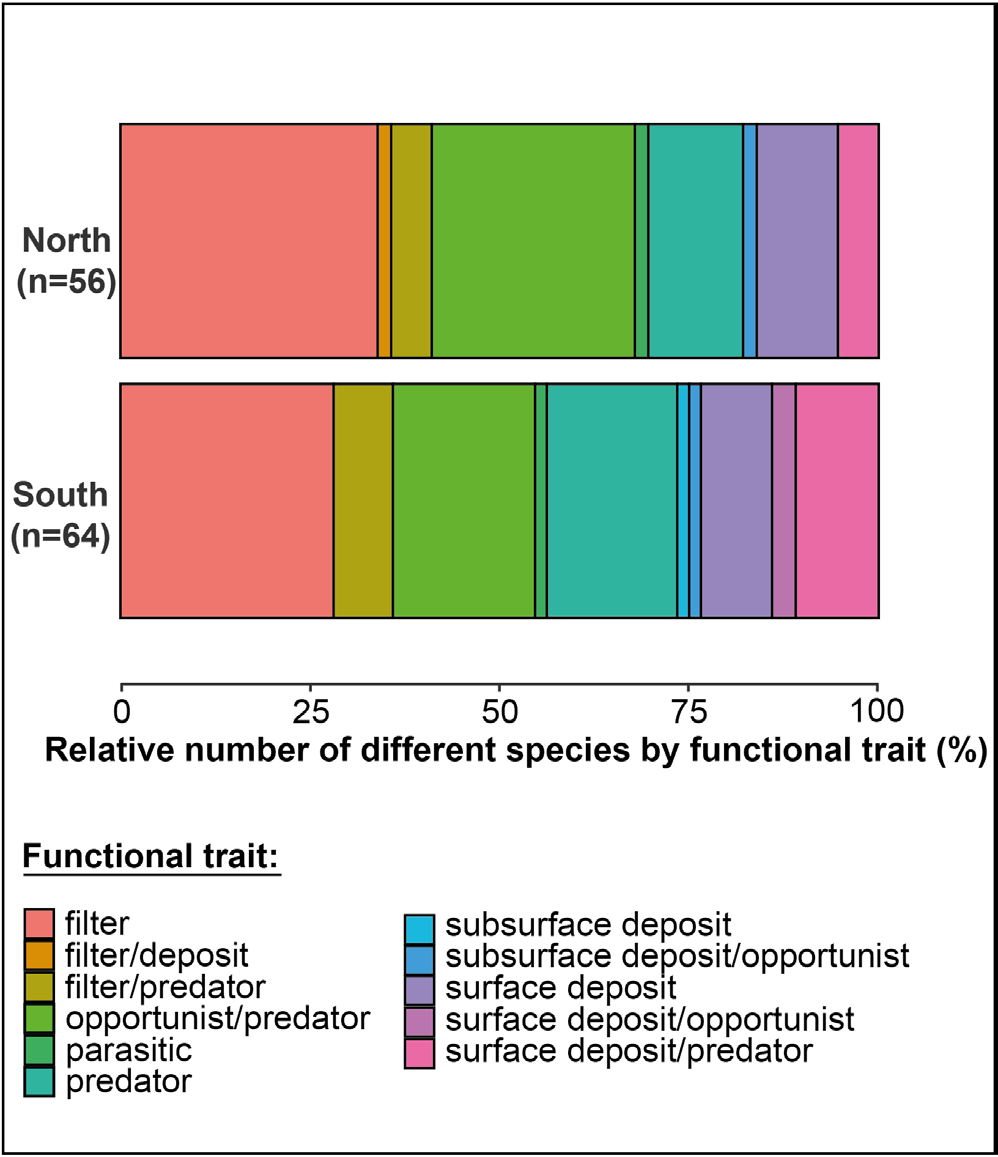
Relative number of different benthic invertebrate species by functional traits based on taxon life‐history characteristics in the northern and southern Southampton Island marine food webs.

Using functional traits defined from established life‐history characteristics, the functional traits with the highest relative number of different species in the northern region were filter feeders (33.9%, *n* = 19), opportunists/predators (26.8%, *n* = 15), and predators (12.5%, *n* = 7). In contrast, the relative functional diversity in the south was primarily composed of decapods (19.4%, *n* = 12), amphipods (14.5%, *n* = 9), and gastropods (9.6%, *n* = 6) (Figure 5). Notably, the predator feeder traits represented a larger proportion in the north (50.0%, *n* = 28) compared to the south (37.5%, *n* = 24). Conversely, the deposit feeder traits were more prevalent in the south (26.5%, *n* = 17) than in the north (19.6%, *n* = 11). Overall, the distribution of species across functional traits was more balanced in the south, which also hosted a greater number of species (64) compared to the north (56), indicating higher functional diversity (Figure 5).

## 4 Discussion

This study revealed, for the first time, that local environmental heterogeneity, driven by fine‐scale gradients, can significantly shape differences in benthic organism trophic position, carbon source utilization, resource pathways, and functional composition in an Arctic coastal ecosystem. Spatial variation in bathymetry, primary production supply, and sea ice concentration were likely key drivers of contrasting food web metrics between the northern and southern regions of Southampton Island. In the north, benthic invertebrates exhibited a wider range of trophic positions, with sea stars occupying the top predator role (TP = 4.8), and relied predominantly on phytoplankton‐derived carbon (SC % = 13.5 ± 10.7), likely due to deeper waters, higher phytoplankton production and lower sympagic production. Indeed, during the summer 2019, phytoplankton production in the northwest region of Southampton Island, which encompassed Frozen Strait, Naujaat (Repulse Bay), and northern Roes Welcome Sound, was 3 times greater than that of Hudson Bay along the southern coast of Southampton Island (Kitching et al. 2026). In the shallower southern region, benthic invertebrates occupied a smaller range of trophic positions (maximum TP = 3.7 ± 0.1, in *Sclerocrangon boreas* ) and had a greater reliance on ice algae (SC% = 35.1 ± 19.7). Correspondingly, filter feeders were more prevalent in the north, where phytoplankton production was higher, whereas the southern zoobenthos, more dependent on sympagic carbon, included more deposit‐feeding taxa.

### 4.1 Depth, Diet, and Ice Cover: North–South Differences in Benthic Sub‐Webs Around Southampton Island

In the northern region, the broad δ^15^N range and relatively narrow δ^13^C values (excluding sea spider; Figure 2) indicate a food web supported by a limited number of basal carbon sources but extending across multiple trophic levels. This chain‐like benthic food‐web structure reaches high trophic levels, such as megafaunal sea stars (TP up to 4.8; Amiraux, Mundy, et al. 2023). In contrast, the southern region displays a narrower δ^15^N range, indicative of a shorter trophic structure, but a wider spread in δ^13^C values, reflecting a greater diversity of basal carbon inputs.

Among the different benthic taxonomic groups investigated, the trophic positions were generally higher in the north compared to the south (Figure 3), which could be attributed to relative differences in bathymetry where depth is greater in the north. Roy et al. (2015), who compared stable isotope signatures of benthic food webs across shelf (≤ 200 m) and slope (≥ 200 m) environments in the Canadian Arctic Archipelago, found that δ^15^N, and thus TP, increased with depth among primary consumers due to enhanced degradation of settling organic matter. However, they also reported a decrease in TP for upper‐level benthic consumers at greater depths, as these predators shifted toward omnivory in response to reduced prey availability. In our study, all benthic taxonomic groups exhibited higher TPs in the northern region, including upper‐level consumers, an observation that may initially seem inconsistent with Roy et al. (2015). However, the mean depth in the north (132.0 m) falls within the shelf zone, where prey availability remains sufficient. Slightly more degraded organic matter likely raises the TP of primary consumers through isotopic enrichment, while sustained prey abundance allows higher‐level predators to maintain carnivorous diets. This combination likely accounts for the consistently elevated TPs observed across trophic levels in the north.

The calculation of the sympagic carbon percentage, which indicates the percentage of sea ice‐derived carbon in an organism, across different benthic taxonomic groups revealed that northern benthic sub‐web rely significantly less on ice algae compared to the southern benthic food web (SC% values of 13.5% ± 10.7% vs. 35.1% ± 19.7%, Figure 4; Table S3). These sympagic carbon values are notably high in both cases, considering that ice algae production represents a variable and relatively low contribution to total annual net primary production (phytoplankton + ice algae) on productive Arctic shelves (1%–26%, Legendre et al. 1992; Payne et al. 2021). Ice algae produce significantly more extracellular polymeric substances than phytoplankton, enhancing their sinking rate (Krembs and Engel 2001; Niemi et al. 2024). The ice algae production period, which occurs in early spring, often coincides with reduced grazing degradation (Nadaï et al. 2021), allowing a more efficient portion of the sympagic primary production to be exported to the seafloor. Ice algae are also generally less sensitive to bacterial remineralisation than phytoplankton during their transport to the seabed (Amiraux, Bonin, et al. 2021; Rontani et al. 2022). Consequently, the relative contribution of ice algae to the primary production reaching the seafloor typically increases with depth (Yunda‐Guarin et al. 2020). In the present study, we observed an opposite trend, with sympagic carbon percentages generally higher in southern benthic organisms inhabiting relatively shallower seafloor depths compared to those in the north. Although the north and south are physically well connected, regional differences in waters‐mass properties induce regional differences in primary production regimes. While sea ice cover is generally lower in the south than in the north, sympagic production is relatively higher in the south due to its seasonal sea ice dynamics (Sibert et al. 2010). Supporting the elevated sympagic input in the south, ice algal chlorophyll *a* concentrations have been reported to reach exceptionally high values of 170 mg m^−2^ near Chesterfield Inlet, just south of Southampton Island (Welch et al. 1991). In contrast, despite the longer persistence of sea ice in summer in the north, this region supports intense pelagic phytoplankton production largely driven by tidal and wind‐mixing along the constricted waterways of Frozen Strait and Roes Welcome Sound (Kitching et al. 2026). Although benthic algae and terrestrial organic matter can substantially fuel food webs in coastal environments, their contributions cannot be reliably quantified with the biomarkers employed in this study because (1) their δ^13^C signatures overlap with those of pelagic or sympagic organic matter (McMeans et al. 2013), and (2) HBIs only resolve the relative inputs of these two microalgal sources (Brown et al. 2014). However, the relatively narrow δ^13^C ranges observed in herbivores, particularly in the northern region (Figure 2), suggest that benthic and terrestrial inputs are minimal relative to the supply from pelagic and sympagic production.

The regional difference in the supply of sympagic organic matter to the benthos is further supported by distinct north/south variations in the species richness of benthic functional traits (Figure 5). In the northern region, a higher number of filter‐feeding species was observed, while deposit‐feeding species were less well represented. Conversely, in the southern region, deposit‐feeding species exhibited greater richness, indicating a shift in dominant feeding strategies between regions. This difference likely reflects the melting of seasonal sea ice in summer, which promotes discontinuous, pulsed sinking and export of ice algae production to the seafloor compared to phytoplankton (Niemi et al. 2024). Consequently, benthic communities reliant on the sympagic system must efficiently exploit fresh phytodetrital pulses on soft sediment, favoring surface or subsurface deposit‐feeders (Figure 5; Pierrejean et al. 2020). Many of these deposit‐feeding species exhibit plasticity by possessing multiple functional traits, likely allowing them to switch behaviors depending on the nature and availability of organic matter, thereby further enhancing their adaptability in such dynamic environments.

### 4.2 Spatial Differences in Pelagic Sub‐Web Metrics and Interconnectedness With Benthic‐Associated Species

Pelagic species that comprise pelagic invertebrates, pelagic and demersal fishes, and marine mammals did not show significant differences in trophic position between the two regions, with the exception of marine mammals (Tables 1 and 2, Table S3A; Figures 1 and 3). This consistency likely reflects both the broader spatial mobility of these taxa and the relatively homogeneous nature of pelagic primary production across regions. Unlike benthic species, which are sessile or not very mobile, and thus strongly influenced by localized stressors (Jørgensen et al. 2019), pelagic consumers are more spatially dynamic and may integrate isotopic signals over wider areas (Bouchard et al. 2015; Stern et al. 2024; Watt et al. 2017). Moreover, the composition of pelagic invertebrate and fish communities was relatively similar between the two regions, suggesting that analogous species occupy comparable trophic niches. This overlap in species identity and feeding strategies likely contributes to the observed uniformity in trophic positions within the pelagic sub‐web. The observed difference in marine mammal trophic positions is primarily attributable to regional differences in the species represented in our sampling. In the northern region, samples included ringed seals, narwhals, and belugas, species commonly found in areas such as the Roes Welcome Sound polynya, Naujaat (Repulse Bay), and Foxe Channel (Loewen et al. 2020; Watt and Hornby 2020). In contrast, only Atlantic walrus were sampled in the southern region, where they are known to occur at higher densities (Hammill et al. 2016). This taxonomic distinction is ecologically significant: unlike the predominantly piscivorous or higher‐trophic‐level species sampled in the north, walrus are benthivorous secondary consumers that rely almost exclusively on bivalves (> 80%; Fisher and Stewart 1997; Garde et al. 2018), which explains the lower average trophic position observed for marine mammals in the south.

Sympagic carbon contributes significantly to all major components of the pelagic compartment, with average values of 21.8% ± 17.3% for pelagic invertebrates, 14.6% ± 9.6% for demersal fish, 6.6% ± 3.5% for pelagic fish, and 53.3% ± 22.2% for marine mammals (Amiraux, Mundy, et al. 2023). Within this compartment, the reliance on sympagic carbon is highly variable, driven by the broader diversity of phyla, taxa and the contrasting feeding strategies among taxonomic groups, particularly among pelagic invertebrates (Figure 4; Tables [Link], [Link], [Link]). Among these, *Calanus hyperboreus* copepods exhibited the highest ice‐derived carbon contributions of all taxonomic groups (including benthic) in the southern region, reaching 94.8% ± 7.3%, compared to 37.2% ± 38.4% in the north. As a key trophic vector in the pelagic sub‐web (Nørregaard et al. 2014; Weydmann et al. 2016), this elevated ice algal contribution suggests a broader reliance on sympagic carbon by numerous pelagic species. This dependency may not be immediately evident at the time of sampling, potentially due to time lags in carbon transfer between trophic levels as well as differences in metabolism across taxa. HBI turnover in an organism depends on its metabolic rate, so lower trophic level species integrate diet over shorter periods than higher trophic level consumers (see Koch et al. 2020 and references herein). Nonetheless, the clear sympagic signal observed in summer in the keystone low trophic level *Calanus hyperboreus* copepods highlights the ecological importance of ice‐algal production in sustaining pelagic ecosystem functioning.

In contrast, Kohlbach et al. (2023) reported a limited contribution of ice‐derived carbon to zooplankton diets under sea ice in the northwestern Barents Sea, attributed to the dominance of pelagic production in that region. This discrepancy likely reflects lower ice algae production in the Barents Sea compared to the Canadian Archipelago (Fuglestad et al. 2020; Wassmann et al. 2006), as well as differences in food web structure and organic matter retention between marine waters from Southampton Island and the Barents Sea. These findings underscore the regional and seasonal complexity of Arctic trophic pathways and the influence of local environmental conditions on carbon flow.

Supporting this spatial contrast, pelagic fish in our study also showed higher sympagic carbon contributions in the south, particularly capelin ( *Mallotus villosus* ), which exhibited 11.4% ± 2.9% in the south versus 4.9% ± 3.6% in the north (Tables S1 and S2). This likely results from the consumption of sympagic carbon‐enriched prey such as copepods. While capelin populations in the Barents Sea have been shown to benefit from enhanced pelagic production under reduced sea‐ice conditions (Stige et al. 2019), our results suggest that around Southampton Island, ice algae‐derived organic matter still contributes appreciably to their diet. Demersal fish, by contrast, exhibited a north–south pattern opposite to that observed in most other taxonomic groups, with higher sympagic carbon contributions in the north than in the south (Tables S1 and S2). This possibly reflects differences in species composition and sample sizes (8 in the north and 18 in the south; Tables S1 and S2). Marine mammals also showed higher ice‐derived carbon values in the north, but the underlying drivers differ. This pattern is primarily linked to differences in species identity, with northern marine mammals integrating dietary signals over broad spatial scales, whereas walruses in the south forage locally near haul‐outs (Solovyova et al. 2024; Watt and Hornby 2020).

As the northern benthic community exhibited lower sympagic carbon percent coupled with higher TP (Figures 3 and 4; Table S3) compared to the south, we tested the relationship between these variables (Figure S2). The results suggest that the proportion of sympagic carbon in an organism is not dependent on its trophic position. In the north, the benthic compartment hosts a longer food chain among invertebrates, up to four trophic levels with top predator sea stars. In contrast, the southern benthic compartment displays a more truncated food chain, limited to three trophic levels and lacking higher trophic level benthic invertebrate predators (Figures 2 and 3). We attribute the structural and functional differences observed in the benthic sub‐web to variations in water masses and bathymetry. These environmental differences likely modulate the sympagic‐pelagic‐benthic connectivity across regions, resulting in a predominantly bottom‐up control in the northern region and a top‐down control in the southern region. Indeed, the southern region, characterized by its shallower seafloor, provides favorable conditions for the establishment of one of the largest walrus haulout sites in the area, with approximately 7000 individuals surveyed in 2017 (Mosnier et al. 2023). This species, which generally feeds at depths of between 6 and 32 m and can dive to depths of around 450 m (Jeanniard‐du‐Dot and Guinet 2021), is a voracious benthic feeder that can consume approximately 57 kg of bivalve biomass per day, which represents 4.7% of its total body mass (Born et al. 2003). Based on an adjusted population of 4900 adult walruses (excluding the ca. 30% of calves and juveniles out of the original 7000 individuals) and an estimated average weight of 735 kg per adult (ca. 900 kg for males and ca. 570 kg for females; COSEWIC 2017), the Walrus Island colony could consume at least 170 tons of clams per day in the southern region of Southampton Island. We suggest that this significant top‐down control exerted by walrus on herbivorous benthic species results in resource competition between benthic invertebrates and Atlantic walrus that prevents benthic top predators (sea stars) from thriving, leading to a more truncated food chain in southern versus northern Southampton Island. This assumption is supported by Fukuyama and Oliver (1985), who proposed that although walruses and sea stars target different bivalve size classes, walrus predation on large bivalves may indirectly affect sea stars by reducing bivalve fecundity and recruitment. This form of apparent competition and mesopredator suppression limits prey availability for sea stars, rendering the benthic sub‐web unsuitable for their persistence and reinforcing trophic truncation. Additionally, walruses, being more specialized bivalve feeders than sea stars (Fisher and Stewart 1997; Garde et al. 2018), may alleviate predation pressure on other benthic species. Acting in concert with the flexible food supply in the southern area, this mechanism likely supports the observed increase in richness of benthic functional traits despite a simplified trophic structure (Figure 5). Yet walrus presence, bathymetry, and spatial variations in food source supply are tightly coupled in this system, hindering any clear separation of walrus predation from other environmental drivers. The higher functional‐trait richness observed in the southern region therefore likely reflects the combined influence of these correlated drivers rather than any single mechanism.

More broadly, the coexistence of distinct food web configurations at relatively small spatial scales, such as the deeper, more complete and bottom‐up controlled benthic sub‐web in the north and the shallower, truncated benthic sub‐web shaped by walrus top‐down pressure in the south, suggests that despite the proximity, northern and southern Southampton Island are functionally distinct. Environmental gradients, particularly depth and food supply, act as partial barriers to the sessile species that comprise the benthic habitat as evidenced by the north versus south differences in functional composition (Figure 5). This leads to the formation of localized food web niches. In this spatial mosaic, habitat‐coupling or mobile generalist predators (including fish) can traverse these environmental gradients and connect otherwise distinct food web systems. Their ability to exploit multiple energy channels across adjacent but functionally distinct food web niches likely contributes to the overall stability and resilience of the Arctic ecosystem (Feng et al. 2025). To strengthen Arctic conservation, it is therefore essential to preserve the diversity of food web niches and the key roles of habitat coupling species and mobile generalist predators. These elements underpin ecological resilience by linking habitats and stabilizing energy flows, enabling the system to better withstand environmental change.

## 5 Conclusion

We observed differences in structure, function, and sympagic‐pelagic‐benthic coupling between the northern and southern marine regions around Southampton Island that underscore the critical influence of spatial variability on Arctic marine ecosystems. Specifically, the deeper northern region supports a complex and bottom‐up controlled benthic sub‐web predominantly reliant on phytoplankton‐derived carbon and hosting top predator sea stars. In contrast, the shallower southern region features a truncated benthic structure shaped by intense Atlantic walrus predation. This top‐down control indirectly competes with sea stars, suppressing their populations. Since Atlantic walruses are more specialized feeders, primarily targeting large bivalves, their selective pressure differs from the broader predatory impact of sea stars, allowing a wider range of benthic organisms to flourish. Combined with the availability of both ice algae and phytoplankton, this pelagic top‐down control contributes to a locally higher richness in benthic functional diversity, despite the simplified trophic structure. With declining sea ice and reduced ice‐algal supply, the functional traits of the southern benthic community are expected to shift toward those observed in the north. Although walrus depend on sea ice for haulout, their predation pressure will likely remain strong in the south because of its shallow, accessible coastal habitats. Consequently, the truncated structure of the southern benthic food web is expected to persist. However, over longer timescales, the loss of ice‐algal primary production, which currently provides nearly half of the carbon supporting both benthic and pelagic top predators, makes ecosystem trajectories more uncertain, as it is likely to drive broad reorganization of Arctic food‐web structure and functioning.

All told, these findings underscore the central role of benthic invertebrates as sentinels of food niches in the Arctic food web. This information highlights the need to implement appropriate conservation strategies that take into account food web niches within Arctic environments, as well as species habitat coupling and mobile predators that connect different niches, which can then be used to monitor food web integrity (Machado et al. 2021). Preserving these unique ecological niches will not only strengthen the overall resilience of the ecosystem but will also ensure the stability of Arctic marine ecosystems more broadly in a context of ongoing environmental change.

## Author Contributions

**Rémi Amiraux:** conceptualization (lead), funding acquisition (supporting), investigation (lead), visualization (lead), writing – review and editing (lead). **C. J. Mundy:** funding acquisition (lead), project administration (supporting), resources (supporting), writing – original draft (supporting). **Marie Pierrejean:** investigation (supporting), visualization (supporting), writing – original draft (supporting). **Philippe Massicotte:** formal analysis (supporting), writing – original draft (supporting). **Steven H. Ferguson:** validation (supporting), writing – original draft (supporting). **Aaron T. Fisk:** validation (supporting), writing – original draft (supporting). **Les N. Harris:** writing – original draft (supporting). **Kevin J. Hedges:** validation (supporting), writing – original draft (supporting). **Kelsey F. Johnson:** formal analysis (supporting), writing – original draft (supporting). **Andrea Niemi:** validation (supporting), writing – original draft (supporting). **Bruno Rosenberg:** formal analysis (supporting), writing – original draft (supporting). **Wesley R. Ogloff:** validation (supporting), writing – original draft (supporting). **Cortney A. Watt:** validation (supporting), writing – original draft (supporting). **David J. Yurkowski:** conceptualization (supporting), funding acquisition (supporting), project administration (supporting), supervision (supporting), writing – original draft (supporting).

## Funding

This work was supported by the Marine Environmental Observation, Prediction and Response Network of Centres of Excellence (MEOPAR‐NCE), Government of Nunavut, Fisheries and Sealing Division, Canadian Northern Economic Development Agency (CanNor), DFO Emerging Fisheries Fund, Polar Knowledge Canada, and a National Science and Engineering Research Council of Canada (NSERC) ship time grant for the Southampton Island Marine Ecosystem Project (SIMEP), a NSERC‐matched grant for the 2017–2018 Belmont Forum and BiodivERsA joint call for research proposals under the BiodivScen ERA‐Net CO‐FUND program to the project ACCES: De‐icing of Arctic Coasts: Critical or new opportunities for marine biodiversity and Ecosystem Services? Individual NSERC Discovery Grant and Northern Research Supplements were provided to RA, CJM and DJY. This research was also funded in part by the Agence Nationale de la Recherche (ANR), grant “ANR‐25‐CE01‐4070”. Several grants from Fisheries and Oceans Canada including the Nunavut Implementation Fund and Strategic Program for Ecosystem‐based Research and Advice were provided to DJY. The Sentinel North program as well as the Transforming Climate Action research program of Université Laval also contributed to this study through salary support for P. Massicotte, made possible in part thanks to funding from the Canada First Research Excellence Fund. Furthermore, this study was supported in part by the Churchill Marine Observatory (CMO), which was funded by the Canada Foundation for Innovation and other partners, including the Arctic Research Foundation (ARF). This work represents a contribution to the Canada Excellence Research Chair (CERC) unit at the University of Manitoba.

## Conflicts of Interest

The authors declare no conflicts of interest.

## Supporting information


**Figure S1:** Boxplots illustrating (A) mean sea ice concentration from June to July between 2016 and 2019, and (B) bathymetry at station locations sampled in both northern and southern regions. Statistical analysis of regional differences was performed using Wilcoxon rank‐sum tests to compare northern and southern areas. *p*‐values resulting from these tests are displayed at the top of each plot.

**Figure S2:** Relationship between sympagic carbon percentage and trophic position of benthic invertebrate collected in the north and south Southampton Island marine food webs Regression lines and their determination coefficients are presented on each graph. Note the use of a logarithmic scale to better visualize the distribution of data points across a wide range of values.

**Table S1:** Carbon and nitrogen stable isotope ratio (δ^13^C and δ^15^N), trophic position (TP), and sympagic carbon (%) of marine food web organisms at the taxonomic group and species level in north Southampton Island.

**Table S2:** Carbon and nitrogen stable isotope ratio (δ^13^C and δ^15^N), trophic position (TP), and sympagic carbon (%) of marine food web organisms at the taxonomic group and species level in South Southampton Island.

**Table S3:** Results of the Wilcoxon test evaluating whether (A) the trophic position is higher in the north than in the south, and (B) the percentage of sympagic carbon is lower in the north than in the south, across various taxonomic phyla or groups. Significant *p*‐values are highlighted in bold.

## Data Availability

All metadata are available on the SIMEP project website: https://open.canada.ca/data/en/dataset/2a4ad7be‐d4b7‐11ee‐bd12‐d17b9d44bf6a.
