# Supplementary figures and images for: Diverging Food Web Functioning Around Southampton Island, Nunavut: The Influence of Primary Production Supply and Bathymetry
Source: Ecol Evol. 2026 Apr 15;16(4):e73448. doi: 10.1002/ece3.73448 (PMC13083602; doi:10.1002/ece3.73448)

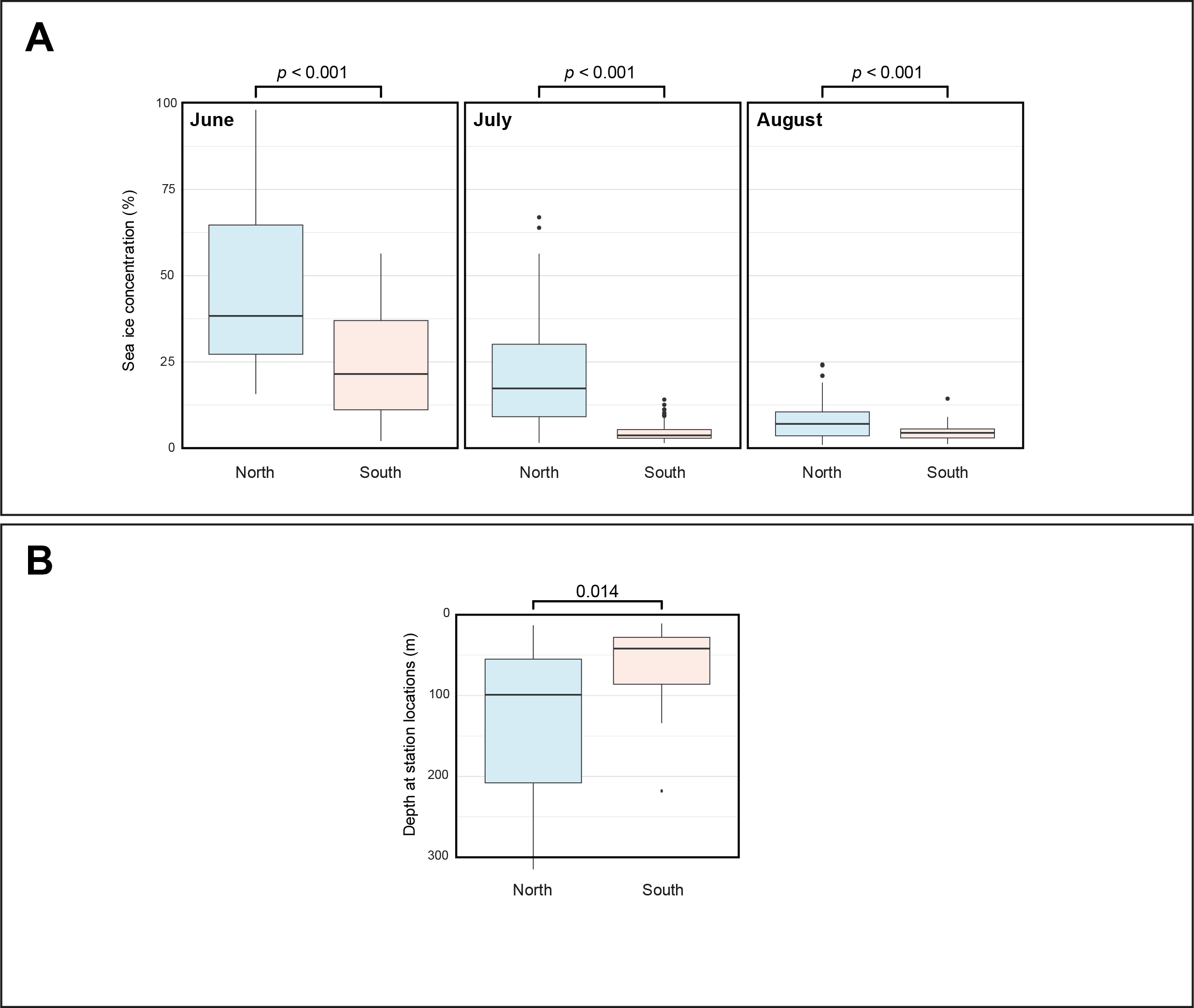

Supplement: Supplementary file 1 — Figure S1: Boxplots illustrating (A) mean sea ice concentration from June to July between 2016 and 2019, and (B) bathymetry at station locations sampled in both northern and southern regions. Statistical analysis of regional differences was performed using Wilcoxon rank‐sum tests to compare northern and southern areas. p‐values resulting from these tests are displayed at the top of each plot. [file ECE3-16-e73448-s005.png]

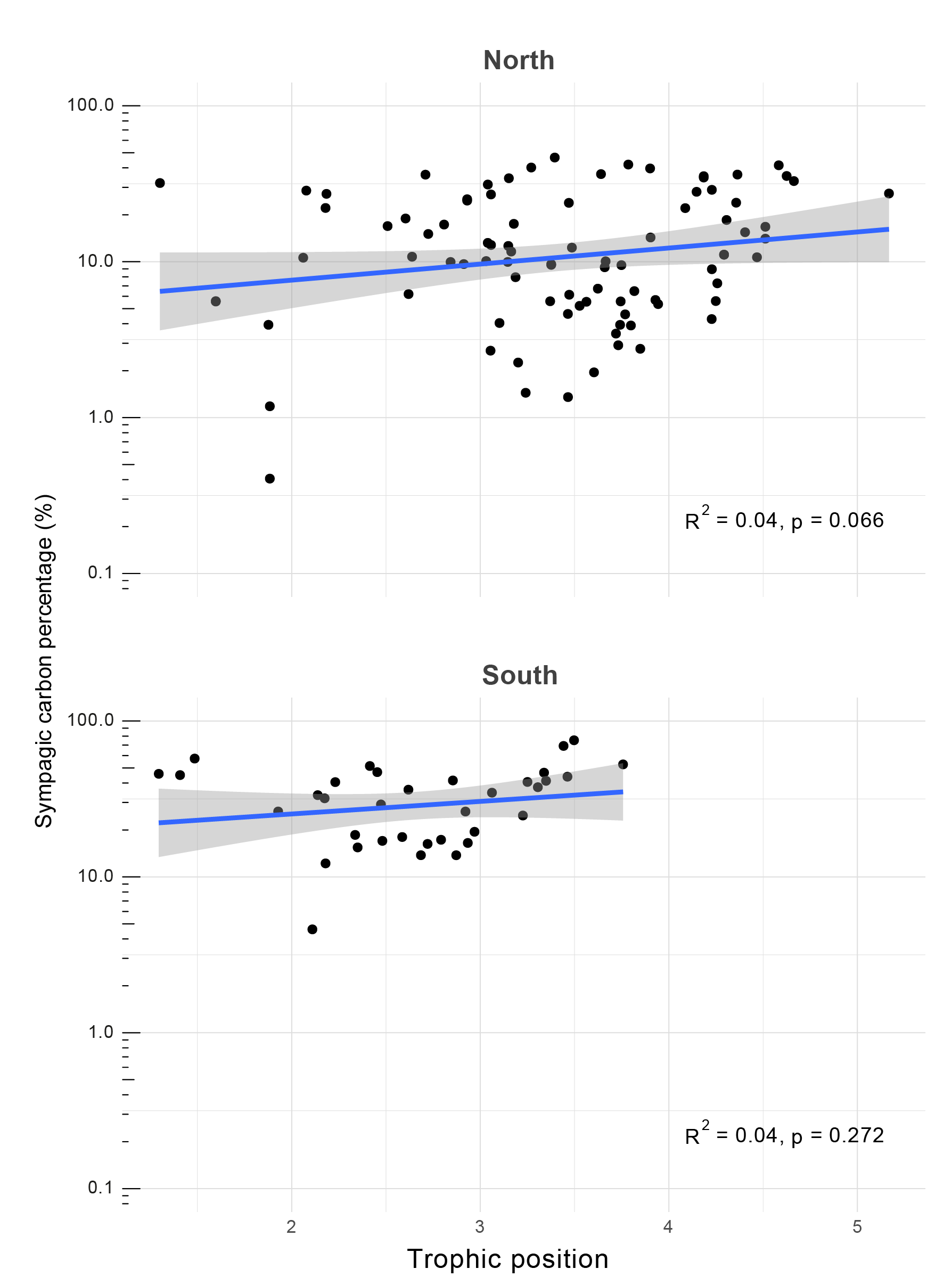

Supplement: Supplementary file 2 — Figure S2: Relationship between sympagic carbon percentage and trophic position of benthic invertebrate collected in the north and south Southampton Island marine food webs Regression lines and their determination coefficients are presented on each graph. Note the use of a logarithmic scale to better visualize the distribution of data points across a wide range of values. [file ECE3-16-e73448-s003.png]
