# Supplementary material for: Diverging Food Web Functioning Around Southampton Island, Nunavut: The Influence of Primary Production Supply and Bathymetry
Source: Ecol Evol. 2026 Apr 15;16(4):e73448. doi: 10.1002/ece3.73448 (PMC13083602; doi:10.1002/ece3.73448)
Supplement: Supplementary file 3 — Table S1: Carbon and nitrogen stable isotope ratio (δ13C and δ15N), trophic position (TP), and sympagic carbon (%) of marine food web organisms at the taxonomic group and species level in north Southampton Island. [file ECE3-16-e73448-s001.docx]

**Table S1:** Carbon and nitrogen stable isotope ratio (δ^13^C and δ^15^N), trophic position (TP), and sympagic carbon (%) of marine food web organisms at the taxonomic group and species level in north Southampton Island.

| **Taxonomic phyla** | **Taxonomic group / specie** | **Tissue** | **n sampled** | **n** | **δ13C (‰)** | **δ15N (‰)** | **TP** | **n** | **Sympagic C (%)** |
| --- | --- | --- | --- | --- | --- | --- | --- | --- | --- |
| **Benthic invertebrate** |  | **Whole organism, soft part, piece of muscle** | **617** | **339** | **-17.1 ± 1.9** | **12.8 ± 2.8** | **3 ± 0.8** | **205** | **13.5 ± 10.7** |
|  | **Amphipod** | **Whole** | **87** | **31** | **-16.9 ± 0.8** | **12.8 ± 1.8** | **3 ± 0.5** | **14** | **14.7 ± 9.3** |
|  | *Anonyx*sp |  | 27 | 16 | -16.8 ± 0.8 | 14.1 ± 1.2 | 3.3 ± 0.4 | 11 | 11.4 ± 7.3 |
|  | *Eusirus cuspidatus* |  | 3 | 3 | -16.9 ± 0.4 | 13.0 ± 0.6 | 3.0 ± 0.2 | *-* | *-* |
|  | *Haploops tubicula* |  | 1 | 1 | -18.5 | 9.8 | 2.1 | *-* | *-* |
|  | *Paramphithoe hystrix* |  | 6 | 6 | -17.2 ± 0.7 | 10.6 ± 0.5 | 2.3 ± 0.2 | *-* | *-* |
|  | *Rhachotropis aculeata* |  | 29 | 5 | -16.3 ± 0.4 | 11.6 ± 0.4 | 2.6 ± 0.1 | 3 | 26.7 ± 5.2 |
|  | **Anthozoan** | **Whole** | **26** | **22** | **-14.9 ± 1.5** | **12.1 ± 1.3** | **2.8 ± 0.4** | **14** | **10.9 ± 7.8** |
|  | *Actiniaria*sp |  | 1 | 1 | -18.2 | 14.5 | 3.5 | 1 | 1.4 |
|  | *Gersemia*sp |  | 21 | 21 | -14.7 ± 1.3 | 12.0 ± 1.2 | 2.7 ± 0.4 | 11 | 12.1 ± 8.2 |
|  | *Neptheidae* |  | 4 | *-* | *-* | *-* | *-* | 2 | 9.0 ± 0.5 |
|  | **Bivalve** | **Soft part** | **46** | **26** | **-18.8 ± 0.7** | **9.1 ± 1.8** | **1.9 ± 0.5** | **4** | **13.6 ± 3.3** |
|  | *Chlamys islandicus* |  | 1 | 1 | -18.6 | 9.6 | 2.0 | *-* | *-* |
|  | *Ciliatocardium ciliatum* |  | 1 | 1 | -20.4 | 7.5 | 1.4 | *-* | *-* |
|  | *Ennucula tenuis* |  | 2 | 2 | -18.3 ± 0.2 | 9.6 ± 0.2 | 2.0 ± 0.1 | *-* | *-* |
|  | *Hiatella arctica* |  | 2 | 2 | -18.8 ± 0.5 | 9.5 ± 0.5 | 2.0 ± 0.2 | *-* | *-* |
|  | *Musculus*sp |  | 3 | 3 | -19.3 ± 0.1 | 8.5 ± 0.3 | 1.7 ± 0.1 | *-* | *-* |
|  | *Nuculana pernula* |  | 2 | 2 | -19.3 ± 0.1 | 7.7 ± 0.4 | 1.5 ± 0.1 | *-* | *-* |
|  | *Nuculana*sp |  | 2 | 2 | -18.7 ± 0.3 | 8.1 ± 1 | 1.6 ± 0.3 | *-* | *-* |
|  | *Pectinidae* |  | 1 | 1 | -17.0 | 11.2 | 2.5 | 1 | 16.9 |
|  | *Serripes groenlandicus* |  | 2 | 2 | -17.8 ± 0.2 | 12.4 ± 2.6 | 2.9 ± 0.8 | *-* | *-* |
|  | *Similipecten greenlandicus* |  | 23 | 3 | -18.4 ± 0.1 | 11.3 ± 0.1 | 2.5 ± 0 | 3 | 12.5 ± 3.1 |
|  | *Bivalvia* |  | 7 | 7 | -19.0 ± 0.4 | 7.9 ± 1.1 | 1.5 ± 0.3 | *-* | *-* |
|  | **Brachiopod** | **Soft part** | **7** | **6** | **-17.9 ± 0.9** | **11.5 ± 0.5** | **2.6 ± 0.1** | ***-*** | ***-*** |
|  | *Hemithiris psittacea* |  | 7 | 6 | -17.9 ± 0.9 | 11.5 ± 0.5 | 2.6 ± 0.1 | *-* | *-* |
|  | **Brittle star** | **Piece** | **17** | **8** | **-18.5 ± 1.6** | **10.0 ± 1.3** | **2.1 ± 0.4** | **6** | **14.0 ± 6.3** |
|  | *Ophiopholis aculeata* |  | 17 | 8 | -18.5 ± 1.6 | 10.0 ± 1.3 | 2.1 ± 0.4 | 6 | 14.0 ± 6.3 |
|  | **Crinoid** | **Piece** | **14** | **11** | **-19.2 ± 1.5** | **13.6 ± 1.4** | **3.2 ± 0.4** | **12** | **10.1 ± 3.1** |
|  | *Heliometra glacialis* |  | 14 | 11 | -19.2 ± 1.5 | 13.6 ± 1.4 | 3.2 ± 0.4 | 12 | 10.1 ± 3.1 |
|  | **Decapod** | **Muscle** | **168** | **64** | **-17.4 ± 0.7** | **14.4 ± 1** | **3.4 ± 0.3** | **70** | **6.0 ± 3.3** |
|  | *Argis dentata* |  | 29 | 10 | -16.9 ± 0.6 | 15.0 ± 1.2 | 3.6 ± 0.4 | 21 | 5.8 ± 2.9 |
|  | *Eualus gaimardii* |  | 39 | 11 | -18.5 ± 0.5 | 13.7 ± 0.6 | 3.2 ± 0.2 | 11 | 5.3 ± 3.8 |
|  | *Lebbeus groenlandicus* |  | 4 | 1 | -17.1 | 14.6 | 3.5 | 1 | 12.3 |
|  | *Lebbeus polaris* |  | 38 | 15 | -17.7 ± 0.4 | 13.7 ± 1 | 3.2 ± 0.3 | 13 | 5.4 ± 2.6 |
|  | *Sabinea septemcarinata* |  | 13 | 1 | -16.3 | 15.4 | 3.7 | 3 | 11.1 ± 7.3 |
|  | *Sclerocrangon boreas* |  | 23 | 16 | -16.8 ± 0.4 | 15.1 ± 0.5 | 3.6 ± 0.2 | 13 | 5.5 ± 2.8 |
|  | *Spirontocaris spinus* |  | 22 | 10 | -17.4 ± 0.3 | 14.7 ± 0.4 | 3.5 ± 0.1 | 8 | 6.3 ± 1.6 |
|  | **Gastropod** | **Soft part** | **29** | **28** | **-17.5 ± 0.5** | **11.8 ± 1.5** | **2.7 ± 0.5** | **3** | **9.9 ± 0.4** |
|  | *Colus*sp |  | 2 | 2 | -17.7 ± 0.3 | 12.3 ± 0.2 | 2.8 ± 0.1 | *-* | *-* |
|  | *Gastropoda* |  | 4 | 4 | -17.7 ± 0.6 | 11.5 ± 0.7 | 2.6 ± 0.2 | *-* | *-* |
|  | *Margarites groenlandicus* |  | 3 | 3 | -17.6 ± 0.5 | 10.2 ± 0.7 | 2.2 ± 0.2 | *-* | *-* |
|  | *Margarites helicinus* |  | 2 | 2 | -17.8 ± 0 | 11.3 ± 0.6 | 2.5 ± 0.2 | *-* | *-* |
|  | *Margarites olivaceous* |  | 11 | 11 | -17.4 ± 0.5 | 11.0 ± 0.5 | 2.4 ± 0.1 | *-* | *-* |
|  | *buccinum*sp |  | 7 | 6 | -17.1 ± 0.6 | 14.4 ± 0.8 | 3.4 ± 0.2 | 3 | 9.9 ± 0.4 |
|  | **Isopod** | **Whole** | **33** | **14** | **-18.1 ± 0.7** | **9.9 ± 0.6** | **2.1 ± 0.2** | **9** | **23.9 ± 3.1** |
|  | *Arcturus baffini* |  | 33 | 14 | -18.1 ± 0.7 | 9.9 ± 0.6 | 2.1 ± 0.2 | 9 | 23.9 ± 3.1 |
|  | **Sea cucumber** | **Whole** | **22** | **17** | **-17.4 ± 1.6** | **11 ± 1.5** | **2.4 ± 0.4** | **6** | **6.6 ± 4.2** |
|  | *Cucumaria frondosa* |  | 8 | 3 | -20.1 ± 0.5 | 8.3 ± 0.2 | 1.6 ± 0.1 | 4 | 5.1 ± 0.9 |
|  | *Molpadia* sp |  | 13 | 13 | -16.5 ± 0.6 | 11.7 ± 0.5 | 2.6 ± 0.1 | 1 | 15.1 |
|  | *holothuroidea* |  | 1 | 1 | -19.8 | 9.1 | 1.9 | 1 | 3.9 |
|  | **Sea spider** | **Piece** | **32** | **11** | **-23.8 ± 0.5** | **11.1 ± 0.9** | **2.5 ± 0.3** | **10** | **22.4 ± 11.8** |
|  | *Boreonymphon abyssorum* |  | 2 | 2 | -23.9 ± 0.2 | 12.1 ± 2 | 2.8 ± 0.6 | 1 | 17.5 |
|  | *Nymphonidae* |  | 30 | 9 | -23.8 ± 0.6 | 10.9 ± 0.5 | 2.4 ± 0.1 | 9 | 23.0 ± 12.3 |
|  | **Sea star** | **Piece** | **27** | **21** | **-17.9 ±1.6** | **16.9 ± 1.6** | **4.2 ± 0.5** | **15** | **16.4 ± 9.8** |
|  | *Asteroidea* |  | 1 | 1 | -21.1 | 19.2 | 4.8 | *-* | *-* |
|  | *Crossaster papposus* |  | 3 | 2 | -16.6 ± 0.3 | 15.5 ± 0.8 | 3.7 ± 0.2 | 1 | 14.3 |
|  | *Diplopteraster multipes* |  | 5 | 4 | -17.2 ± 0.9 | 18 ± 0.5 | 4.5 ± 0.1 | 4 | 17.1 ± 2.2 |
|  | *Henricia* sp |  | 4 | 4 | -16.8 ± 1.1 | 17.1 ± 0.7 | 4.2 ± 0.2 | 2 | 35.0 ± 0.5 |
|  | *Pteraster militaris* |  | 7 | 6 | -19.1 ± 1.7 | 16.4 ± 2.1 | 4.0 ± 0.6 | 3 | 14.8 ± 6.3 |
|  | *Pteraster pulvillus* |  | 3 | *-* | *-* | *-* | *-* | 1 | 25.3 |
|  | *Solaster*sp |  | 4 | 4 | -18.0 ± 1.4 | 16.3 ± 1.5 | 4.0 ± 0.4 | 4 | 6.0 ± 3.4 |
|  | **Sea urchin** | **Piece** | **24** | **12** | **-19.2 ± 0.9** | **9.1 ± 0.7** | **1.9 ± 0.2** | **12** | **8.0 ± 4** |
|  | *Strongylocentrotus droebachiensis* |  | 24 | 12 | -19.2 ± 0.9 | 9.1 ± 0.7 | 1.9 ± 0.2 | 12 | 8.0 ± 4 |
|  | **Sponge** | **Whole** | **37** | **35** | **-17.9 ± 1.7** | **16.2 ± 2.7** | **4.0 ± 0.8** | **19** | **29.1 ± 10.4** |
|  | *Porifera* |  | 37 | 35 | -17.9 ± 1.7 | 16.2 ± 2.7 | 4.0 ± 0.8 | 19 | 29.1 ± 10.4 |
|  | **Worms** | **Whole** | **46** | **33** | **-17.6 ± 1.1** | **12.6 ± 2** | **2.9 ± 0.6** | **11** | **30.1 ± 9.8** |
|  | *Eunoe oerstedi* |  | 9 | 9 | -17.4 ± 0.5 | 13.8 ± 0.8 | 3.3 ± 0.2 | 5 | 35.6 ± 8.6 |
|  | *Polychaeta* |  | 37 | 24 | -17.7 ± 1.3 | 12.1 ± 2.1 | 2.8 ± 0.6 | 6 | 25.4 ± 8.7 |
| **Pelagic invertebrate** |  | **Whole** | **256** | **122** | **-19.8 ± 0.7** | **10.9 ± 1.6** | **2.4 ± 0.5** | **29** | **12.3 ± 17.8** |
|  | **Amphipod** |  | **67** | **24** | **-19.3 ± 0.9** | **11.0 ± 1.4** | **2.4 ± 0.4** | **7** | **1.8 ± 1.1** |
|  | *Hyperia medusarum* |  | 4 | 2 | -19.5 ± 1.3 | 11.9 ± 1.6 | 2.7 ± 0.5 | *-* | *-* |
|  | *Themisto abyssorum* |  | 6 | 6 | -19.7 ± 0.5 | 12.9 ± 0.5 | 3.0 ± 0.2 | *-* | *-* |
|  | *Themisto libellula* |  | 53 | 16 | -19.1 ± 1 | 10.2 ± 0.8 | 2.2 ± 0.2 | 7 | 1.8 ± 1.1 |
|  | **Chaetognathan** |  | **26** | **21** | **-19.4 ± 0.2** | **13.4 ± 0.4** | **3.1 ± 0.1** | **5** | **11.7 ± 3.4** |
|  | *Chaetognatha* |  | 26 | 21 | -19.4 ± 0.2 | 13.4 ± 0.4 | 3.1 ± 0.1 | 5 | 11.7 ± 3.4 |
|  | **Copepod** |  | **25** | **21** | **-20.1 ± 0.4** | **9.8 ± 0.6** | **2.1 ± 0.2** | **4** | **37.2 ± 38.4** |
|  | *Calanus hyperboreus* |  | 18 | 14 | -19.8 ± 0.2 | 9.5 ± 0.3 | 2.0 ± 0.1 | 4 | 37.2 ± 38.4 |
|  | *Metridia*sp |  | 7 | 7 | -20.5 ± 0.4 | 10.5 ± 0.4 | 2.3 ± 0.1 | *-* | *-* |
|  | **Ctenophore** |  | **4** | ***-*** | ***-*** | ***-*** | ***-*** | **2** | **2.8 ± 2.6** |
|  | *Ctenophora* |  | 4 | *-* | *-* | *-* | *-* | 2 | 2.8 ± 2.6 |
|  | **Hydrozoan** |  | **14** | **7** | **-20.0 ± 0.9** | **11.8 ± 1** | **2.7 ± 0.3** | **4** | **3.2 ± 4.1** |
|  | *Hydrozoa* |  | 14 | 7 | -20.0 ± 0.9 | 11.8 ± 1 | 2.7 ± 0.3 | 4 | 3.2 ± 4.1 |
|  | **Krill and Mysid** |  | **113** | **44** | **-19.8 ± 0.4** | **10.1 ± 0.9** | **2.2 ± 0.3** | **7** | **16.7 ± 8.9** |
|  | *Meganyctiphanes norvegica* |  | 3 | 3 | -19.9 ± 0.1 | 10.7 ± 0.5 | 2.4 ± 0.1 | *-* | *-* |
|  | *Mysis oculata* |  | 26 | 15 | -19.9 ± 0.3 | 9.7 ± 1 | 2.0 ± 0.3 | 2 | 15.3 ± 1.2 |
|  | *Thysanoessa inermis* |  | 17 | 10 | -20.0 ± 0.3 | 10.7 ± 1.1 | 2.3 ± 0.3 | 2 | 13.5 ± 5.4 |
|  | *Thysanoessa raschii* |  | 67 | 16 | -19.7 ± 0.4 | 9.9 ± 0.5 | 2.1 ± 0.2 | 3 | 19.8 ± 13.9 |
|  | **Pteropod** |  | **7** | **5** | **-21.4 ± 0.8** | **10.3 ± 2.3** | **2.2 ± 0.7** | ***-*** | ***-*** |
|  | *Clione limacina* |  | 5 | 3 | -21.2 ± 0.1 | 11.7 ± 1.7 | 2.6 ± 0.5 | *-* | *-* |
|  | *Pteropoda* |  | 2 | 2 | -21.8 ± 1.5 | 8.1 ± 0.1 | 1.6 ± 0 | *-* | *-* |
| **Demersal fish** |  | **Muscle** | **61** | **61** | **-18.5 ± 0.9** | **14.4 ± 1.5** | **3.4 ± 0.4** | **8** | **21.5 ± 9.3** |
|  | *Apidophoroides olrikii* |  | 12 | 12 | -18.6 ± 0.4 | 14.5 ± 1 | 3.5 ± 0.3 | *-* | *-* |
|  | *Eumesogrammus praecisus* |  | 12 | 12 | -17.5 ± 1 | 14.1 ± 1.8 | 3.3 ± 0.5 | 2 | 13.1 ± 0 |
|  | *Icelus bicornis* |  | 4 | 4 | -19.0 ± 0.4 | 14.2 ± 1.7 | 3.4 ± 0.5 | *-* | *-* |
|  | *Icelus spatula* |  | 2 | 2 | -18.9 ± 0.6 | 14.1 ± 2.1 | 3.3 ± 0.6 | *-* | *-* |
|  | *Leptagonus decagonus* |  | 2 | 2 | -19.1 ± 1.3 | 16.3 ± 1.2 | 4.0 ± 0.3 | *-* | *-* |
|  | *Leptoclinus maculatus* |  | 1 | 1 | -19.2 | 12.8 | 3.0 | *-* | *-* |
|  | *Myoxocephalus scorpius* |  | 6 | 6 | -17.7 ± 0.8 | 15.9 ± 0.8 | 3.9 ± 0.2 | 6 | 24.3 ± 9.2 |
|  | *Triglops pingelii* |  | 22 | 22 | -19.1 ± 0.4 | 14.1 ± 1.3 | 3.3 ± 0.4 | *-* | *-* |
| **Pelagic fish** |  | **Muscle** | **114** | **77** | **-19.9 ± 0.7** | **14.1 ± 1.5** | **3.3 ± 0.4** | **72** | **7.4 ± 7.7** |
|  | Arctic cod |  | 51 | 44 | -19.8 ± 0.7 | 14.7 ± 1.1 | 3.5 ± 0.3 | 30 | 9.9 ± 10.8 |
|  | Capelin |  | 49 | 19 | -20.0 ± 0.9 | 12.4 ± 1.3 | 2.9 ± 0.4 | 30 | 4.9 ± 3.6 |
|  | Polar cod |  | 14 | 14 | -19.9 ± 0.6 | 14.6 ± 1 | 3.5 ± 0.3 | 12 | 7.8 ± 2.5 |
| **Marine mammal** |  | **Muscle** |  | **68** | **-18.2 ± 0.3** | **16.8 ± 1.5** | **4.5 ± 0.6** | **64** | **53.5 ± 24.6** |
|  | Beluga |  |  | 12 | -18.2 ± 0.3 | 16.0 ± 0.5 | 4.2 ± 0.2 | 8 | 31.0 ± 28.3 |
|  | Narwhal |  |  | 15 | -18.1 ± 0.3 | 15.4 ± 1.2 | 3.9 ± 0.5 | 11 | 40.1 ± 12.0 |
|  | Ringed Seal |  |  | 41 | -18.3 ± 0.4 | 17.6 ± 1.3 | 4.8 ± 0.5 | 45 | 60.7 ± 22.8 |
