# Supplementary material for: Diverging Food Web Functioning Around Southampton Island, Nunavut: The Influence of Primary Production Supply and Bathymetry
Source: Ecol Evol. 2026 Apr 15;16(4):e73448. doi: 10.1002/ece3.73448 (PMC13083602; doi:10.1002/ece3.73448)
Supplement: Supplementary file 4 — Table S2: Carbon and nitrogen stable isotope ratio (δ13C and δ15N), trophic position (TP), and sympagic carbon (%) of marine food web organisms at the taxonomic group and species level in South Southampton Island. [file ECE3-16-e73448-s002.docx]

**Table S2:** Carbon and nitrogen stable isotope ratio (δ^13^C and δ^15^N), trophic position (TP), and sympagic carbon (%) of marine food web organisms at the taxonomic group and species level in South Southampton Island.

|  |  |  |  |  |  |  |  |  |  |
| --- | --- | --- | --- | --- | --- | --- | --- | --- | --- |
| **Taxonomic phyla** | **Taxonomic group / specie** | **Tissue** | **n sampled** | **n** | **δ^13^C (‰)** | **δ^15^N (‰)** | **TP** | **n** | **Sympagic C (%)** |
| **Benthic invertebrate** |  | **Whole organism, soft part, piece of muscle** | 1253 | 541 | -18.4 ± 2.3 | 11.3 ± 2.2 | 2.5 ± 0.6 | 260 | 35.1 ± 19.7 |
|  | **Amphipod** | **Whole** | **92** | **40** | **-17.6 ± 1.5** | **11.4 ± 2.5** | **2.6 ± 0.7** | **16** | **26.1 ± 17.9** |
|  | *Anonyx*sp |  | 27 | 16 | -17.0 ± 1.1 | 13.2 ± 2 | 3.1 ± 0.6 | 11 | 31.8 ± 16.9 |
|  | *Caprella septentrionalis* |  | 4 | 4 | -19.5 ± 0.6 | 7.4 ± 0.3 | 1.4 ± 0.1 | *-* | *-* |
|  | *Halirages fulvocinctus* |  | 5 | 5 | -18.8 ± 0.6 | 9.5 ± 0.2 | 2.0 ± 0.1 | *-* | *-* |
|  | *Ischyrocerus anguipes* |  | 3 | 3 | -20.1 ± 0.7 | 8.3 ± 0.8 | 1.6 ± 0.2 | *-* | *-* |
|  | *Oediceros borealis* |  | 1 | 1 | -17.9 | 12.5 | 2.9 | *-* | *-* |
|  | *Rhachotropis aculeata* |  | 38 | 10 | -16.6 ± 0.5 | 11.6 ± 0.7 | 2.6 ± 0.2 | 5 | 13.5 ± 13.9 |
|  | *Stegocephalus inflatus* |  | 1 | 1 | -15.5 | 15.0 | 3.6 | *-* | *-* |
|  | **Anthozoan** | **Whole** | **11** | **9** | **-18.6 ± 1.5** | **9.8 ± 1.3** | **2.1 ± 0.4** | **2** | **10.1 ± 9.7** |
|  | *Gersemia*sp |  | 9 | 9 | -18.6 ± 1.5 | 9.8 ± 1.3 | 2.1 ± 0.4 | 1 | 17.0 |
|  | *Neptheidae* |  | 2 | *-* | *-* | *-* | *-* | 1 | 3.2 |
|  | **Ascidiacea** | **Whole** | **49** | **41** | **-22.2 ± 0.8** | **9.8 ± 1.6** | **2.1 ± 0.5** | **8** | **26.7 ± 3.2** |
|  | *Ascidiacea* |  | 8 | 8 | -21.7 ± 0.5 | 7.2 ± 1.6 | 1.3 ± 0.5 | *-* | *-* |
|  | *Boltenia echinata* |  | 6 | 6 | -21.4 ± 1 | 10.3 ± 1.1 | 2.2 ± 0.3 | *-* | *-* |
|  | *Boltenia ovifera* |  | 14 | 14 | -22.2 ± 0.5 | 10.5 ± 0.8 | 2.3 ± 0.2 | *-* | *-* |
|  | *Boltenia villosa* |  | 21 | 13 | -23 ± 0.5 | 10.5 ± 0.5 | 2.3 ± 0.2 | 8 | 26.7 ± 3.2 |
|  | **Barnacle** | **Soft part** | **36** | **25** | **-18.9 ± 0.7** | **8.3 ± 0.6** | **1.7 ± 0.2** | **11** | **58.1 ± 3.5** |
|  | *Balanus*sp |  | 36 | 25 | -18.9 ± 0.7 | 8.3 ± 0.6 | 1.7 ± 0.2 | 11 | 58.1 ± 3.5 |
|  | **Bivalve** |  | **27** | **22** | **-20.5 ± 0.6** | **7.8 ± 1.3** | **1.5 ± 0.4** | ***-*** | ***-*** |
|  | *Crenella* sp |  | 8 | 8 | -20.2 ± 0.2 | 6.5 ± 0.2 | 1.1 ± 0.1 | *-* | *-* |
|  | *Hiatella arctica* |  | 12 | 9 | -20.8 ± 0.6 | 8.8 ± 0.6 | 1.8 ± 0.2 | *-* | *-* |
|  | *Musculus discors* |  | 2 | 2 | -20.7 ± 1.1 | 7.3 ± 1.5 | 1.3 ± 0.4 | *-* | *-* |
|  | *Pectinidae* |  | 1 | 1 | -20.6 | 8.7 | 1.8 | *-* | *-* |
|  | *bivalvia* |  | 2 | 2 | -19.6 ± 0 | 8.1 ± 2.5 | 1.6 ± 0.7 | *-* | *-* |
|  | **Brittle star** | **Piece** | **205** | **30** | **-18.5 ± 1.5** | **9.4 ± 1.3** | **2.0 ± 0.4** | **20** | **40.2 ± 18.3** |
|  | *Ophiacantha bidentata* |  | 5 | 3 | -17.5 ±0.8 | 10.9 ± 0.3 | 2.4 ± 0.1 | *-* | *-* |
|  | *Ophiopholis aculeata* |  | 200 | 27 | -18.6 ±1.5 | 9.2 ± 1.2 | 1.9 ± 0.4 | 20 | 40.2 ± 18.3 |
|  | **Bryozoan** | **Piece** | **9** | **2** | **-18.3 ± 4.7** | **7.6 ± 0.2** | **1.4 ± 0.1** | **9** | **44.9 ± 14.7** |
|  | *Bryozoa* |  | 8 | 1 | -21.6 | 7.8 | 1.5 | 8 | 44.8 ± 15.7 |
|  | *Eucratea*sp |  | 1 | 1 | -14.9 | 7.5 | 1.4 | 1 | 45.0 |
|  | **Chiton** | **Soft part** | **19** | **10** | **-19.9 ± 1.3** | **11.0 ± 1.1** | **2.4 ± 0.3** | ***-*** | ***-*** |
|  | *Boreochiton ruber* |  | 5 | 5 | -20.7 ± 1.4 | 10.9 ± 0.4 | 2.4 ± 0.1 | *-* | *-* |
|  | *Stenosemus albus* |  | 1 | 1 | -19.0 | 10.1 | 2.2 | *-* | *-* |
|  | *Tonicella marmoreus* |  | 1 | 1 | -20.7 | 9.2 | 1.9 | *-* | *-* |
|  | *Tonicella*sp |  | 12 | 3 | -18.8 ± 0.2 | 12.2 ± 0.7 | 2.8 ± 0.2 | *-* | *-* |
|  | **Crinoid** | **Piece** | **14** | **10** | **-20.9 ± 1.2** | **11.7 ± 1** | **2.6 ± 0.3** | **13** | **29.2 ± 26.2** |
|  | *Crinoid* sp |  | 3 | *-* | *-* | *-* | *-* | 3 | 73.0 ± 11 |
|  | *Heliometra glacialis* |  | 11 | 10 | 20.9 ±1.2 | 11.7 ± 1 | 2.6 ± 0.3 | 10 | 16.0 ± 7.6 |
|  | **Decapod** | **Muscle** | **579** | **216** | **-17.9 ± 1.4** | **12.6 ± 1.4** | **2.9 ± 0.4** | **124** | **31.4 ± 19.1** |
|  | *Argis dentata* |  | 54 | 19 | -14.9 ± 1.1 | 14.3 ± 0.7 | 3.4 ± 0.2 | 13 | 39.3 ± 32.3 |
|  | *Eualus fabricii* |  | 86 | 20 | -18.1 ± 1.2 | 12.5 ± 0.7 | 2.9 ± 0.2 | 13 | 24.4 ± 8.8 |
|  | *Eualus gaimardii* |  | 101 | 43 | -17.6 ± 1 | 12.0 ± 0.7 | 2.7 ± 0.2 | 25 | 29.8 ± 13.8 |
|  | *Hyas coarctatus* |  | 13 | 11 | -18.4 ± 1.5 | 13.7 ± 0.8 | 3.2 ± 0.2 | 7 | 43.0 ± 27.9 |
|  | *Lebbeus groenlandicus* |  | 51 | 12 | -17.6 ± 0.4 | 13.2 ± 0.6 | 3.1 ± 0.2 | 17 | 32.3 ± 17.5 |
|  | *Lebbeus polaris* |  | 167 | 62 | -18.5 ± 0.9 | 11.9 ± 1.4 | 2.7 ± 0.4 | 24 | 26.2 ± 15 |
|  | *Pagurus*sp |  | 2 | 2 | -19.3 ± 0 | 12.9 ± 0.7 | 3.0 ± 0.2 | *-* | *-* |
|  | *Pandalus montagui* |  | 34 | 11 | -18.1 ± 0.5 | 14.0 ± 0.8 | 3.3 ± 0.2 | 5 | 11.0 ± 13.6 |
|  | *Sabinea septemcarinata* |  | 3 | *-* | *-* | *-* | *-* | 1 | 13.8 |
|  | *Sclerocrangon boreas* |  | 5 | 5 | -17.0 ± 0.5 | 15.4 ± 0.5 | 3.7 ± 0.1 | 1 | 52.7 |
|  | *Spirontocaris phippsi* |  | 15 | 8 | -19.1 ± 0.5 | 12.4 ± 0.8 | 2.9 ± 0.2 | 2 | 37.1 ± 3.8 |
|  | *Spirontocaris spinus* |  | 48 | 23 | -18.3 ± 0.6 | 11.7 ± 1.2 | 2.6 ± 0.4 | 16 | 40.4 ± 16.2 |
|  | **Gastropod** | **Soft part** | **87** | **59** | **-20 ± 1.1** | **11.6 ± 1.6** | **2.6 ± 0.5** | **20** | **39 ± 19.6** |
|  | *Dendronotus*sp |  | 52 | 28 | -20.6 ± 0.4 | 12.9 ± 0.5 | 3.0 ± 0.1 | 18 | 42.8 ± 16.6 |
|  | *Littorinidae* |  | 14 | 13 | -20.2 ± 0.9 | 10.0 ± 0.4 | 2.1 ± 0.1 | *-* | *-* |
|  | *Margarites helicinus* |  | 10 | 10 | -18.2 ± 1.1 | 9.5 ± 0.9 | 2.0 ± 0.3 | *-* | *-* |
|  | *Velutinidae* |  | 4 | 4 | -20.5 ± 0.3 | 11.6 ± 0.6 | 2.6 ± 0.2 | *-* | *-* |
|  | *buccinum*sp |  | 6 | 4 | -18.6 ± 0.3 | 13.1 ± 0.8 | 3.0 ± 0.2 | 2 | 4.7 ± 1.2 |
|  | **Isopod** | **Whole** | **13** | **10** | **-18.5 ± 0.3** | **13.3 ± 0.5** | **3.1 ± 0.1** | ***-*** | ***-*** |
|  | *Bopyroides hippolytes* |  | 13 | 10 | -18.5 ± 0.3 | 13.3 ± 0.5 | 3.1 ± 0.1 | *-* | *-* |
|  | **Sea cucumber** | **Whole** | **9** | **7** | **-18.2 ± 1.7** | **10.3 ± 1.8** | **2.2 ± 0.5** | ***-*** | ***-*** |
|  | *Cucumaria frondosa* |  | 5 | 4 | -19.2 ± 0.3 | 10.9 ± 2.3 | 2.4 ± 0.7 | *-* | *-* |
|  | *Psolus fabricii* |  | 3 | 3 | -16.9 ± 2.2 | 9.6 ± 0.6 | 2.0 ± 0.2 | *-* | *-* |
|  | **Sea star** | **Piece** | **24** | **12** | **-19.3 ± 2.6** | **11.1 ± 1** | **2.5 ± 0.3** | **15** | **34.7 ± 11** |
|  | *Henricia* sp |  | 5 | 3 | -20.9 ±3.6 | 12.2 ± 1.1 | 2.8 ± 0.3 | 5 | 41.1 ± 6 |
|  | *Leptasterias groenlandica* |  | 7 | 6 | -17.7 ±1.3 | 10.9 ± 0.6 | 2.4 ± 0.2 | 5 | 38.2 ± 8.5 |
|  | *Leptasterias (Hexasterias) polaris* |  | 4 | 2 | -20 ±2.5 | 10.0 ± 0.2 | 2.1 ± 0 | 3 | 21.8 ± 14.9 |
|  | *Solaster endeca* |  | 1 | 1 | -22.1 | 11.7 | 2.6 | *-* | *-* |
|  | *Stephanasterias albula* |  | 7 | *-* | *-* | *-* | *-* | 2 | 29.6 ± 2.4 |
|  | **Sea urchin** | **Piece** | **34** | **19** | **-17.8 ± 2.6** | **8.5 ± 2** | **1.7 ± 0.6** | **12** | **52.6 ± 22** |
|  | *Strongylocentrotus droebachiensis* |  | 34 | 19 | -17.8 ± 2.6 | 8.5 ± 2 | 1.7 ± 0.6 | 12 | 52.6 ± 22 |
|  | **Sponge** | **Whole** | **2** | **1** | **-21.0** | **9.3** | **1.9** | **2** | **39.3 ± 18.5** |
|  | *Porifera* |  | 2 | 1 | -21.0 | 9.3 | 1.9 | 2 | 39.3 ± 18.5 |
|  | **Worms** | **Whole** | **39** | **28** | **-20 ± 1.4** | **12.9 ± 1** | **3.0 ± 0.3** | **8** | **44.8 ± 19** |
|  | *Nereis pelagica* |  | 14 | 12 | -20.5 ± 1.8 | 12.9 ± 1.2 | 3.0 ± 0.4 | 1 | 11.4 |
|  | *Polychaeta* |  | 25 | 16 | -19.7 ± 1 | 13 ± 0.8 | 3.0 ± 0.2 | 7 | 49.5 ± 14.4 |
| **Pelagic** |  | **Whole** |  | **181** | **-20.7 ± 1.4** | **10.7 ± 1.4** | **2.4 ± 0.4** | **99** | **26 ± 22** |
| **invertebrate** | **Amphipod** |  | **73** | **38** | **-20.0 ± 0.6** | **11.1 ± 1.3** | **2.5 ± 0.4** | **13** | **19.1 ± 16.6** |
|  | *Hyperia galba* |  | 8 | 8 | -19.7 ± 0.6 | 12.9 ± 0.9 | 3.0 ± 0.3 | *-* | *-* |
|  | *Hyperia medusarum* |  | 2 | 2 | -19.9 ± 0.9 | 12.2 ± 0.3 | 2.8 ± 0.1 | *-* | *-* |
|  | *Themisto libellula* |  | 63 | 28 | -20.1 ± 0.6 | 10.5 ± 0.9 | 2.3 ± 0.3 | 13 | 19.1 ± 16.6 |
|  | **Chaetognathan** |  | **42** | **17** | **-20.6 ± 0.3** | **13.0 ± 0.2** | **3.0 ± 0.1** | **25** | **29.2 ± 25** |
|  | *Chaetognatha* |  | 42 | 17 | -20.6 ± 0.3 | 13.0 ± 0.2 | 3.0 ± 0.1 | 25 | 29.2 ± 25 |
|  | **Copepod** |  | **21** | **19** | **-20.8 ± 1** | **9.8 ± 0.7** | **2.1 ± 0.2** | **2** | **94.8 ± 7.3** |
|  | *Calanus hyperboreus* |  | 16 | 14 | -20.4 ± 0.4 | 9.5 ± 0.4 | 2.0 ± 0.1 | 2 | 94.8 ± 7.3 |
|  | *Metridia*sp |  | 5 | 5 | -21.9 ± 1.5 | 10.7 ± 0.9 | 2.3 ± 0.3 | *-* | *-* |
|  | **Ctenophore** |  | **3** | **3** | **-20 ± 0.1** | **12.0 ± 0.4** | **2.7 ± 0.1** | **2** | **36.7 ± 8.9** |
|  | *Ctenophora* |  | 3 | 3 | -20.0 ± 0.1 | 12.0 ± 0.4 | 2.7 ± 0.1 | 2 | 36.7 ± 8.9 |
|  | **Hydrozoan** |  | **70** | **40** | **-20.0 ± 1.4** | **11.3 ± 0.8** | **2.5 ± 0.2** | **17** | **37.0 ± 17.3** |
|  | *Aglantha*sp |  | 4 | 4 | -19.7 ± 0.2 | 11.6 ± 0.2 | 2.6 ± 0.1 | *-* | *-* |
|  | *Hydroid* |  | 1 | *-* | *-* | *-* | *-* | 1 | 32.7 |
|  | *Hydrozoa* |  | 65 | 36 | -20.0 ± 1.5 | 11.2 ± 0.9 | 2.5 ± 0.3 | 16 | 37.3 ± 17.8 |
|  | **Krill and Mysid** |  | **99** | **29** | **-20.4 ± 0.8** | **9.5 ± 0.9** | **2 ± 0.3** | **14** | **21.0 ± 12.6** |
|  | *Mysis oculata* |  | 16 | 4 | -20.2 ± 0.9 | 9.2 ± 0.6 | 1.9 ± 0.2 | 4 | 14.8 ± 8.7 |
|  | *Stilomysis grandis* |  | 1 | 1 | -19.5 | 13.6 | 3.2 | *-* | *-* |
|  | *Thysanoessa raschii* |  | 82 | 24 | -20.5 ± 0.8 | 9.4 ± 0.5 | 2 ± 0.1 | 10 | 23.5 ± 13.4 |
|  | **Pteropod** |  | **69** | **33** | **-22.8 ± 0.9** | **9.8 ± 1.1** | **2.1 ± 0.3** | **25** | **15.0 ± 15.9** |
|  | *Clione limacina* |  | 42 | 17 | -22.6 ± 0.5 | 10.8 ± 0.4 | 2.4 ± 0.1 | 14 | 20.5 ± 19.7 |
|  | *Limacina helicina* |  | 5 | 5 | -21.4 ± 0.7 | 8.2 ± 0.8 | 1.6 ± 0.2 | *-* | *-* |
|  | *Pteropoda* |  | 22 | 11 | -23.6 ± 0.5 | 9 ± 0.2 | 1.9 ± 0.1 | 11 | 7.9 ± 0.9 |
|  | **Squid** |  | **3** | **2** | **-18.6 ± 0.1** | **13.4 ± 0.6** | **3.1 ± 0.2** | **1** | **38.0** |
|  | *Gonatus*sp |  | 3 | 2 | -18.6 ± 0.1 | 13.4 ± 0.6 | 3.1 ± 0.2 | 1 | 38.0 |
| **Demersal fish** |  | **Muscle** |  | **244** | **-18.9 ± 1.5** | **14.3 ± 1.2** | **3.4 ± 0.4** | **18** | **11.9 ± 11.3** |
|  | *Ammodytes*sp |  | 5 | 5 | -22.4 ± 1.1 | 12.8 ± 0.3 | 3.0 ± 0.1 | *-* | *-* |
|  | *Eumesogrammus praecisus* |  | 17 | 17 | -18.7 ± 0.7 | 15.1 ± 1.3 | 3.6 ± 0.4 | *-* | *-* |
|  | *Gymnelus viridis* |  | 2 | 2 | -19.5 ± 0.2 | 14.9 ± 0.2 | 3.6 ± 0.1 | *-* | *-* |
|  | *Gymnocanthus tricuspis* |  | 34 | 34 | -16.4 ± 0.3 | 13.4 ± 0.8 | 3.1 ± 0.2 | *-* | *-* |
|  | *Icelus spatula* |  | 1 | 1 | -18.6 | 15.1 | 3.6 | *-* | *-* |
|  | *Leptagonus decagonus* |  | 2 | 2 | -19.6 ± 0 | 14.3 ± 1.7 | 3.4 ± 0.5 | *-* | *-* |
|  | *Leptoclinus maculatus* |  | 5 | 5 | -19.6 ± 0.3 | 14.3 ± 0.5 | 3.4 ± 0.1 | *-* | *-* |
|  | *Liparis*sp |  | 2 | 2 | -19.6 ± 0.6 | 12.4 ± 0.7 | 2.8 ± 0.2 | *-* | *-* |
|  | *Lumpenus fabricii* |  | 24 | 24 | -17.4 ± 1 | 13.3 ± 0.9 | 3.1 ± 0.3 | *-* | *-* |
|  | *Myoxocephalus scorpioides* |  | 17 | 15 | -19.0 ± 1.4 | 15 ± 1.2 | 3.6 ± 0.3 | 2 | 20.8 ± 0 |
|  | *Myoxocephalus scorpius* |  | 18 | 18 | -19.3 ± 0.9 | 15.3 ± 1.2 | 3.7 ± 0.4 | 2 | 35.5 ± 11.2 |
|  | *Myoxocephalus* sp |  | 3 | 3 | -19.5 ± 0.8 | 13.8 ± 1.1 | 3.3 ± 0.3 | *-* | *-* |
|  | *Pholis fasciata* |  | 28 | 28 | -19.9 ± 0.6 | 13.8 ± 1.2 | 3.3 ± 0.3 | *-* | *-* |
|  | *Stichaeus punctatus* |  | 37 | 37 | -19.3 ± 0.5 | 15.2 ± 1 | 3.7 ± 0.3 | *-* | *-* |
|  | *Triglops murrayi* |  | 39 | 38 | -19.6 ± 0.8 | 13.8 ± 0.8 | 3.3 ± 0.2 | 2 | 15.2 ± 0 |
|  | *Triglops pingelii* |  | 14 | 9 | -20.1 ± 0.2 | 15.1 ± 0.4 | 3.6 ± 0.1 | 12 | 5.9 ± 5.3 |
|  | *Triglops*sp |  | 3 | 3 | -20.2 ± 0.2 | 15.3 ± 0.3 | 3.7 ± 0.1 | *-* | *-* |
|  | *Zoarcidae*sp |  | 1 | 1 | -19.2 | 14.6 | 3.5 | *-* | *-* |
| **Pelagic fish** |  | **Muscle** | **49** | **44** | **-20.0 ± 0.8** | **14.0 ± 0.9** | **3.3 ± 0.3** | **6** | **10.1 ± 2.9** |
|  | Arctic cod |  | 25 | 24 | -19.8 ± 0.8 | 13.8 ± 1 | 3.2 ± 0.3 | 2 | 7.7 ± 0 |
|  | Capelin |  | 19 | 15 | -20.1 ± 0.9 | 14 ± 0.5 | 3.3 ± 0.1 | 4 | 11.4 ± 2.9 |
|  | Greenland cod |  | 5 | 5 | -20.7 ± 0.1 | 15.4 ± 0.4 | 3.7 ± 0.1 | *-* | *-* |
| **Marine mammal** |  | **Muscle** |  | **4** | **-18.5 ± 0.3** | **12.9 ± 1.0** | **3.0 ± 0.3** | **4** | **44.5 ± 7.8** |
|  | Walrus |  |  | 4 | -18.5 ± 0.3 | 12.9 ± 1.0 | 3.0 ± 0.3 | 4 | 44.5 ± 7.8 |
