# Supplementary material for: Diverging Food Web Functioning Around Southampton Island, Nunavut: The Influence of Primary Production Supply and Bathymetry
Source: Ecol Evol. 2026 Apr 15;16(4):e73448. doi: 10.1002/ece3.73448 (PMC13083602; doi:10.1002/ece3.73448)
Supplement: Supplementary file 5 — Table S3: Results of the Wilcoxon test evaluating whether (A) the trophic position is higher in the north than in the south, and (B) the percentage of sympagic carbon is lower in the north than in the south, across various taxonomic phyla or groups. Significant p‐values are highlighted in bold. [file ECE3-16-e73448-s004.docx]

| Taxonomic phyla |  | n | *p-value* | statistic |
| --- | --- | --- | --- | --- |
| Benthic invertebrate | *amphipod* | 71 | **0.02** | 806 |
|  | *anthozoan* | 31 | **0.00** | 175 |
|  | *bivalve* | 48 | **0.00** | 414 |
|  | *brittle star* | 38 | 0.17 | 147 |
|  | *crinoid* | 21 | **0.00** | 99 |
|  | *decapod* | 280 | **0.00** | 12018 |
|  | *gastropod* | 87 | 0.44 | 844 |
|  | *isopod* | 24 | 1.00 | 0 |
|  | *sea cucumber* | 24 | 0.11 | 79 |
|  | *sea star* | 33 | **0.00** | 250 |
|  | *sea urchin* | 31 | **0.01** | 169 |
|  | *sponge* | 36 | 0.06 | 34 |
|  | *worms* | *61* | 0.75 | 415 |
| *pelagic invertebrate* |  | 303 | 0.29 | 11448 |
| *demersal fish* |  | 305 | 0.12 | 8161 |
| *pelagic fish* |  | 121 | 0.26 | 1815 |
| *marine mamals* |  | 141 | **0.01** | 1064 |
|  |  |  |  |  |
|  |  |  |  |  |
|  |  |  |  |  |
| **(B)** |  |  |  |  |
| Taxonomic phyla |  | n | *p_value* | statistic |
| Benthic invertebrate | *amphipod* | 30 | 0.07 | 75 |
|  | *anthozoan* | 16 | 0.53 | 14 |
|  | *brittle star* | 26 | **0.00** | 6 |
|  | *crinoid* | 22 | **0.00** | 18 |
|  | *decapod* | 194 | **0.00** | 464 |
|  | *gastropod* | 23 | **0.01** | 6 |
|  | *sea star* | 30 | **0.00** | 27 |
|  | *sea urchin* | 24 | **0.00** | 0 |
|  | *sponge* | 21 | 0.30 | 14 |
|  | *worms* | 19 | **0.03** | 21 |
| *pelagic invertebrate* |  | 128 | **0.00** | 780 |
| *demersal fish* |  | 26 | 0.98 | 110 |
| *pelagic fish* |  | 78 | **0.01** | 98 |
| *marine mamals* |  | 68 | 0.69 | 146 |

**Table S3:** Results of the Wilcoxon test evaluating whether (A) the trophic position is higher in the north than in the south, and (B) the percentage of sympagic carbon is lower in the north than in the south, across various taxonomic phyla or groups. Significant *p-values* are highlighted in bold.

**(A)**
